# Infection with MERS-CoV Causes Lethal Pneumonia in the Common Marmoset

**DOI:** 10.1371/journal.ppat.1004250

**Published:** 2014-08-21

**Authors:** Darryl Falzarano, Emmie de Wit, Friederike Feldmann, Angela L. Rasmussen, Atsushi Okumura, Xinxia Peng, Matthew J. Thomas, Neeltje van Doremalen, Elaine Haddock, Lee Nagy, Rachel LaCasse, Tingting Liu, Jiang Zhu, Jason S. McLellan, Dana P. Scott, Michael G. Katze, Heinz Feldmann, Vincent J. Munster

**Affiliations:** 1 Disease Modeling and Transmission, Laboratory of Virology, Division of Intramural Research, National Institute of Allergy and Infectious Diseases, National Institutes of Health, Rocky Mountain Laboratories, Hamilton, Montana, United States of America; 2 Rocky Mountain Veterinary Branch, Division of Intramural Research, National Institute of Allergy and Infectious Diseases, National Institutes of Health, Rocky Mountain Laboratories, Hamilton, Montana, United States of America; 3 Department of Microbiology, University of Washington, Seattle, Washington, United States of America; 4 Virus Ecology Unit, Laboratory of Virology, Division of Intramural Research, National Institute of Allergy and Infectious Diseases, National Institutes of Health, Rocky Mountain Laboratories, Hamilton, Montana, United States of America; 5 Department of Immunology and Microbial Science, Department of Integrative Structural and Computational Biology, The Scripps Research Institute, La Jolla, California, United States of America; 6 Department of Biochemistry, Geisel School of Medicine at Dartmouth, Hanover, New Hampshire, United States of America; 7 Department of Medical Microbiology, University of Manitoba, Winnipeg, Manitoba, Canada; Vanderbilt University, United States of America

## Abstract

The availability of a robust disease model is essential for the development of countermeasures for Middle East respiratory syndrome coronavirus (MERS-CoV). While a rhesus macaque model of MERS-CoV has been established, the lack of uniform, severe disease in this model complicates the analysis of countermeasure studies. Modeling of the interaction between the MERS-CoV spike glycoprotein and its receptor dipeptidyl peptidase 4 predicted comparable interaction energies in common marmosets and humans. The suitability of the marmoset as a MERS-CoV model was tested by inoculation via combined intratracheal, intranasal, oral and ocular routes. Most of the marmosets developed a progressive severe pneumonia leading to euthanasia of some animals. Extensive lesions were evident in the lungs of all animals necropsied at different time points post inoculation. Some animals were also viremic; high viral loads were detected in the lungs of all infected animals, and total RNAseq demonstrated the induction of immune and inflammatory pathways. This is the first description of a severe, partially lethal, disease model of MERS-CoV, and as such will have a major impact on the ability to assess the efficacy of vaccines and treatment strategies as well as allowing more detailed pathogenesis studies.

## Author Summary

The development of vaccines and treatment strategies is aided by robust animal disease models that accurately depict the illness that is observed in humans. Here we describe a new, improved model for MERS-CoV using the common marmoset, whereby the severe, and even lethal, illness that has been observed in many human cases is recapitulated. Prior to the development of this model, the only available animal models for MERS-CoV infection were the rhesus macaque and a mouse model that requires adenovirus-transduced expression of the human version of the protein required for virus entry. The rhesus macaque model more closely mimics the mild to moderate disease observed in some patients—mainly those without significant comorbidities. The increased severity of illness in the common marmoset model is an important advance in the ability to evaluate potential therapeutic agents against MERS-CoV, as discrimination between successfully treated and control animals should be more apparent. In addition, the closer models recapitulate the disease observed in humans, the more likely findings can be eventually translated into use in humans.

## Introduction

Since the emergence of MERS-CoV in 2012, researchers have worked to establish animal disease models to study the pathogenesis of this virus and to develop effective countermeasures. With over 836 cases and at least 290 deaths [1], there has been a rapid increase in the number of MERS-CoV cases as diagnostics are being more widely applied. While dromedary camels are suspected to be involved in zoonotic transmission of MERS-CoV [2]–[5]; and camel as well as horse DPP4 can efficiently facilitate virus entry [6], the mechanism(s) by which most people acquire MERS-CoV is still unclear. Since targeted attempts to prevent zoonotic transmission are currently not feasible and significant morbidity and mortality still occur in individuals with comorbidities, developing effective prophylactic and therapeutic treatment strategies remains a high priority. Although several treatment regimens have been suggested for use in patients based on *in vitro* MERS-CoV studies or treatment implemented during the SARS-CoV pandemic, only one such treatment has been tested *in vivo* against MERS-CoV to date [7]. To enable a better evaluation of MERS-CoV treatment and prevention strategies, an animal model more representative of severe human disease is crucial.

Assessment of potential treatments in relevant animal disease models is essential prior to conducting clinical trials. Attempts to generate small animal models of disease have so far not been successful. Mice [8], [9], hamsters [10] and ferrets [11] do not support replication of MERS-CoV in what appears to be a receptor dependent manner. To overcome this impediment, it was recently demonstrated that adenovirus vectored transduction of human DPP4 into multiple strains of mice, including various knock-outs, results in productive infection and mild pneumonia [12]. Notwithstanding the aforementioned mouse models, the only naturally permissive MERS-CoV disease model that has been described to date is the rhesus macaque model [13]–[15]. In the rhesus macaque, MERS-CoV causes a transient infection of the lower respiratory tract resulting in mild to moderate clinical disease. While this model is useful, it seems to recapitulate the mild disease observed in some human cases, rather than the more severe or even lethal disease observed in many human cases. Moreover, although this model has been used to assess treatment strategies [7], [16], it can be difficult as an evaluative model for therapeutics as clinical signs are mild, the duration of illness is relatively short and virus replication is limited.

Dipeptidyl peptidase 4 (DPP4, also known as CD26) was recently shown to be the cellular receptor for MERS-CoV [17], and the interaction between the MERS-CoV spike protein and DPP4 was subsequently determined by co-crystallography studies [18], [19]. Although DPP4 is a relatively conserved protein in general, recent data have shown that receptor specificity is likely a major factor in the species tropism of MERS-CoV [11], [20]. This suggests that mapping the spike binding region of DPP4 of a species of interest before performing experimental inoculations of animals could provide a more rational approach to identifying MERS-CoV susceptible animal models. Here, we modeled the interaction of the common marmoset DPP4 with the MERS-CoV spike protein and show that no differences exist compared to human DPP4 at the site of interaction. Subsequent inoculation of common marmosets (*Callithrix jacchus*) resulted in severe, even lethal, respiratory disease in inoculated animals, with widespread, coalescing bronchointerstitial pneumonia and high viral loads in the lungs of all animals.

## Results

### Common marmoset DPP4 is predicted to bind the MERS-CoV spike glycoprotein

**Figure 1:**
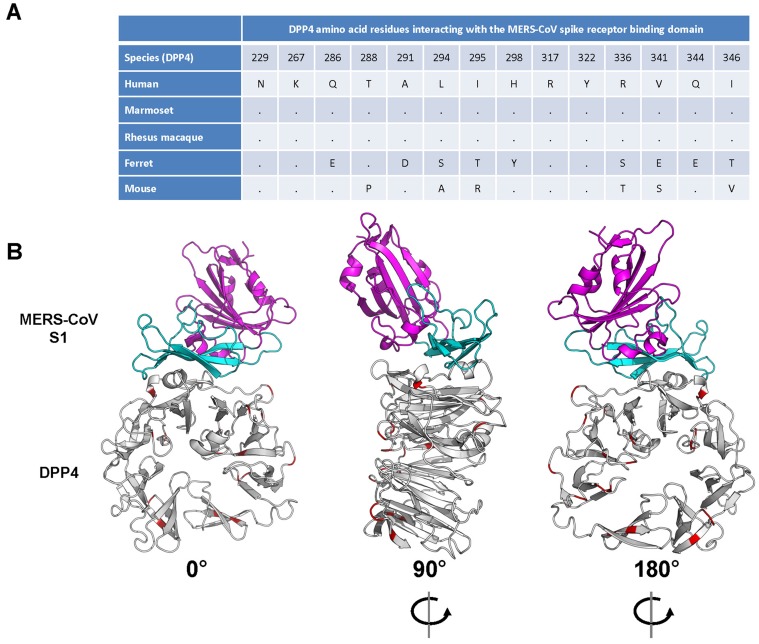
Interaction between the MERS-CoV spike glycoprotein (S1) and its receptor dipeptidyl peptidase 4 (DPP4). (**A**) Alignment of the amino acid residues from human, common marmoset and ferret DPP4 that have been identified to interact with the receptor binding domain of the MERS-CoV spike glycoprotein. (**B**) Interaction model (front, back and side view) of the MERS-CoV S1 and its cognate receptor human DPP4, with amino acid differences in common marmoset DPP4 are highlighted in red.

Variations in the DPP4 receptor appear to play a major role in the ability of MERS-CoV to infect certain animal species. To predict the ability of MERS-CoV spike protein to bind to marmoset DPP4, analyses were performed using human DPP4 (known to bind MERS-CoV spike glycoprotein) [17] and ferret DPP4 (unable to bind MERS-CoV spike glycoprotein) [11]. Comparison of the amino acid alignments of human, ferret and marmoset DPP4 revealed that marmoset DPP4 is 96.4% identical to human DPP4, while ferret DPP4 is 87.4% identical to human and 87.5% identical to marmoset DPP4. Recently, the 14 amino acids in human DPP4 that facilitate binding to the receptor-binding domain (RBD) of the MERS-CoV spike glycoprotein were identified by co-crystallography studies [18], [19]. No amino acid differences between human, rhesus macaque and common marmoset were identified within the DPP4 region interacting with the MERS-CoV RBD, whereas between human/rhesus/marmoset and ferret and mouse nine or six amino acid residues were different within this region, respectively (Fig. 1A). The 100% identity of the 14 amino acid residues between human and marmoset DPP4 in the interaction regions indicates that MERS-CoV RBD should bind to marmoset DPP4. This was further supported by modeling the binding potential between human, ferret and marmoset DPP4 with the MERS-CoV RBD. No significant differences in binding energy were observed between the human (−981) and marmoset (−978) DPP4 – MERS-CoV RBD, whereas the binding energies between ferret DPP4 and the MERS-CoV RBD was significantly higher (−601). A marmoset DPP4 homology model was built using the human DPP4 structure (PDB ID: 4KR0, Chain A). This model demonstrated that all amino acid differences between human and marmoset DPP4 were located away from the binding region of DPP4 with the S1 portion of the spike glycoprotein; the nearest residues to this interface that differ are Arg343, Ile193 and Val279 which are 14A, 10A and 13A away, respectively, from the nearest atom in MERS-S (Fig. 1B). Taken together this suggests that marmoset DPP4 would facilitate binding with the MERS-CoV RBD and that marmosets would be susceptible to MERS-CoV infection.

### Common marmosets develop severe respiratory disease following inoculation with MERS-CoV

**Figure 2:**
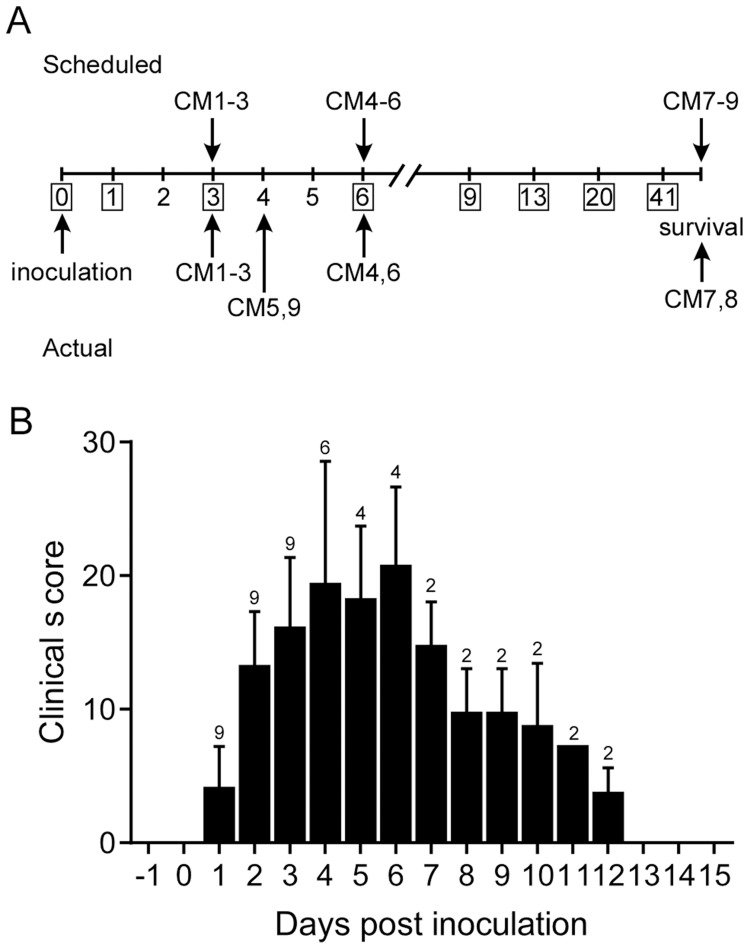
Experimental schedule and clinical parameters. (**A**) Scheduled necropsies are indicated above and actual necropsies below the timeline. CM1–CM9 indicate the study animals. (**B**) Animals were scored daily and their mean clinical score ±SD calculated. The number of animals remaining in the experiment is indicated above the bar on each day.

Common marmosets inoculated with MERS-CoV (hCoV-EMC/2012 [21]) via the intratracheal, intranasal, oral and ocular routes developed signs of respiratory disease that ranged from moderate to severe (Table S1). Animals were assigned for scheduled necropsies on 3 (CM1, CM2 and CM3) and 6 (CM4, CM5 and CM6) dpi prior to the start of the experiment; three additional animals (CM7, CM8 and CM9) were monitored for survival (Fig. 2A). Starting on 1 dpi six of nine animals showed increased respiration rates while all animals had increased respiration rates from 2 dpi on. In some of the animals open mouth and/or labored breathing was evident from 3 dpi onwards. All animals showed loss of appetite and decreased levels of activity. Clinical scores were assigned using an established scoring sheet for respiratory disease in common marmosets (Table S2). Based on this system, peak clinical scores were observed between 4 and 6 dpi with scores returning to baseline by 13 dpi in the two remaining animals (Fig. 2B). Animals exhibited decreased temperatures starting on 3 dpi, returning to a normal range on 9 dpi (Fig. S1A). On 4 dpi, two animals (CM5 and CM9) were euthanized due to the severity of disease as determined by clinical score (Fig. 2A), which included increased respiration rate, open mouth breathing and failure to move following prompting. One of these animals also exhibited the presence of frothy hemorrhagic discharge from its mouth. While most animals showed a decrease in body temperature by 3 dpi; the two animals that were euthanized on 4 dpi were severely hypothermic indicative of the onset of shock at the terminal stages of disease (Fig. S1A). Clinically significant alterations in blood cell counts and chemistry were not noted in any of the animals (Fig. S1B–J) in contrast to the rhesus macaque model. As serial blood samples could not be collected on a daily basis on marmosets, due to the small size of the animals, alterations were likely missed as a result of study design. Elevations in liver enzymes (Fig. S1E–G) and measures of kidney function (Fig. S1I,J) occurred on 3 and 4 dpi, respectively; however, these changes were not outside of the normal range. The animals euthanized on 3, 4 and 6 dpi all showed hypoproteinemia consistent with high protein pulmonary effusions resulting from alveolar edema (Fig. S1K,L).

### Respiratory disease is characterized by progressive severe pneumonia with extensive lung pathology

**Figure 3:**
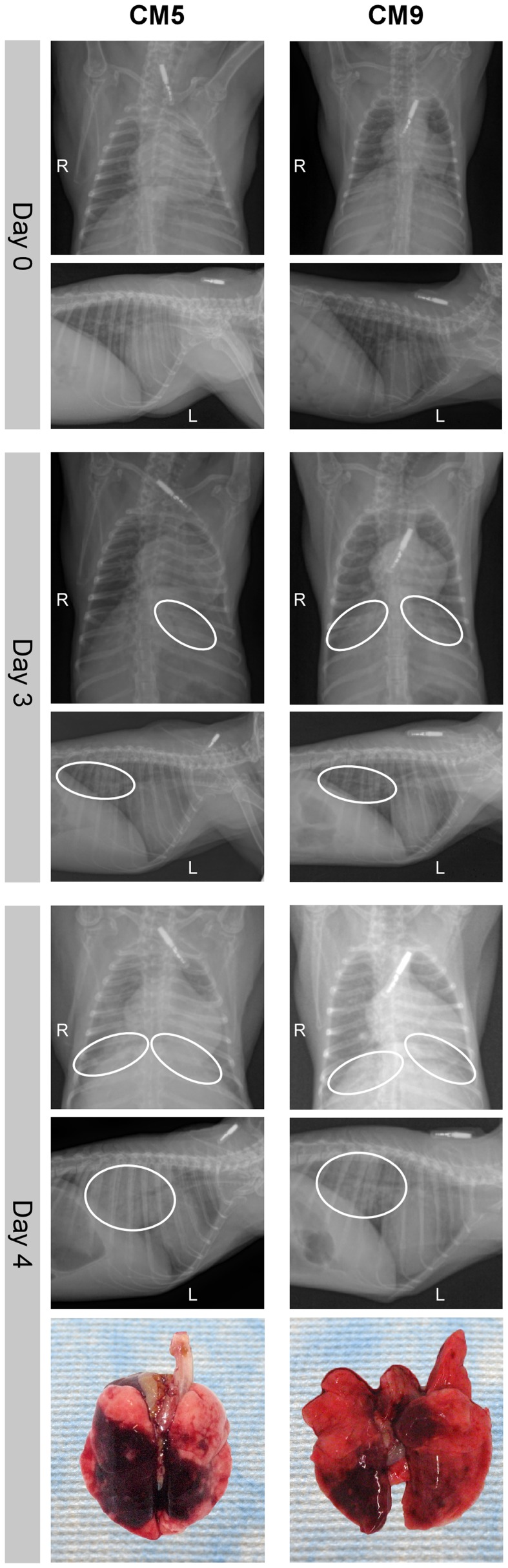
Radiographic alterations and lung pathology. Dorsal-ventral and lateral thoracic x-rays from of common marmosets (CM) imaged prior to MERS-CoV inoculation (day 0) and on days 3 and 4 post-inoculation. Areas of interstitial infiltration, indicative of pneumonia, are highlighted (circle). Positional indicators are included (R – right, L – left). Gross pathology of the lungs from CM5 and CM9 necropsied on 4 dpi are shown below indicating extensive gross lesions. dpi are shown below indicating extensive gross lesions.

To monitor for signs of pneumonia animals underwent dorsal-ventral and lateral x-rays during examinations on 0, 1, 3, 6, 9, 13 and 20 dpi. All radiographs were normal prior to inoculation on 0 dpi, while on 1 dpi four of nine animals showed mild to marked diffuse interstitial infiltration in the lower lung lobes (Table S3). On 3 dpi interstitial infiltration of varying severity (mild to severe) was noted in all animals bilaterally in the lower lobes. The two animals that were euthanized on 4 dpi showed severe interstitial infiltration with partial to complete congestion of the bronchioles (Fig. 3). The other animals were not radiographed at this time point as this was not a scheduled examination time point. On 6 dpi the remaining animals had mild to severe interstitial infiltration in the lower lobes. One animal (CM6) had severe infiltration in all lobes with congested bronchioles. By 9 dpi the two remaining animals showed improvement, with lessening infiltration, which was resolved by 13 dpi.

Three animals were euthanized on 3 dpi and necropsies were performed. All three animals had relatively comparable gross lesions (Fig. S2A), especially in the lower lobes (mean affected area 11% of upper lung lobes, 54% of lower lung lobes), with multifocal consolidation and dark red discoloration, consistent with interstitial pneumonia. The two animals euthanized due to the severity of disease on 4 dpi showed extensive severe lesions throughout the lungs (mean affected area 36% of upper lung lobes, 83% of lower lung lobes) (Fig. 3, Fig. S2A). In addition, the lungs were firm, failed to collapse and were fluid filled. The lungs from animal CM5 were three times the lung weight to body weight ratio of the lungs from the other sampled animals, which were comparable to CM9 (Fig. S2B). Animals necropsied on 6 dpi revealed lungs comparable to 4 dpi with lesions throughout the lungs (mean affected area 12% of upper lung lobes, 85% of lower lung lobes) (Fig. S2A). Lungs from these animals were firm and fluid filled, with fluid leaking from the tissue and had a lung to body weight ratio twice that of 3 dpi animals (Fig. S2B). No other gross pathological changes were observed at necropsy.

### Lungs develop extensive bronchointerstitial pneumonia

**Figure 4:**
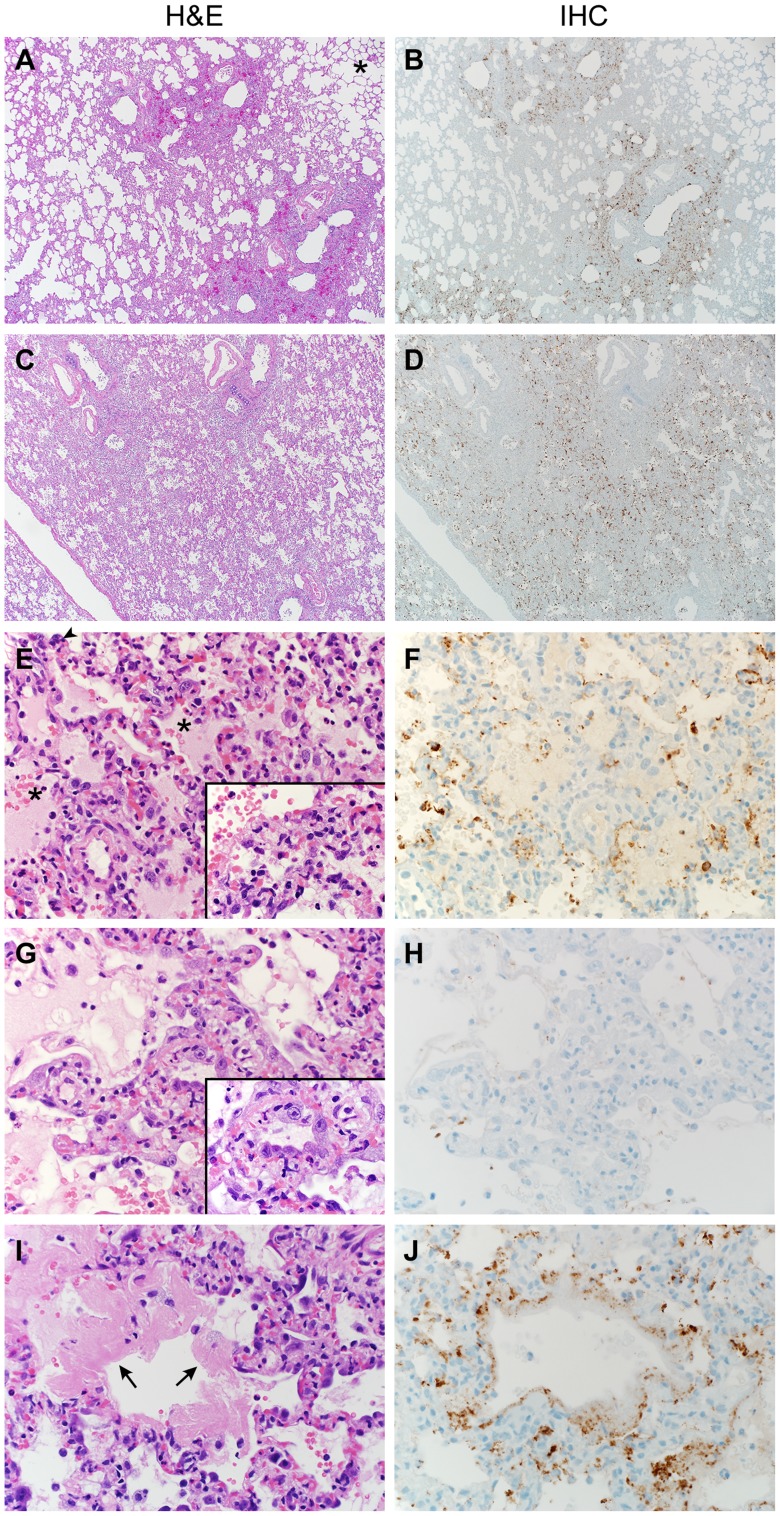
Histopathological changes in lungs of common marmosets inoculated with MERS-CoV. Common marmosets were euthanized on day 3, 4 or 6 post inoculation and lung tissue was collected and stained with hematoxylin and eosin (H&E; panels **A**, **C**, **E**, **G**, **I**) or immunohistochemistry using a polyclonal α-MERS-CoV antibody (IHC; panels **B**, **D**, **F**, **H**, **J**). (**A**) Acute bronchointerstitial pneumonia centered on terminal bronchioles, with influx of inflammatory cells and thickening of alveolar septa in lung tissue collected on 3 dpi. Asterisk indicates essentially normal tissue. (**B**) IHC staining of sequential section of panel A reveals abundance of MERS-CoV antigen in affected areas. (**C**) Coalescing bronchointerstitial pneumonia inducing a diffuse lesion on 3 dpi. (**D**) IHC staining of sequential section of panel C reveals abundance of MERS-CoV antigen in affected areas. (**E**) Edema, hemorrhage and fibrin (asterisks) fill the alveolar spaces in lung tissue collected on 3 dpi. Arrowhead indicates syncytium. Inset highlights thickened alveolar interstitium with fibrin, edema and inflammatory cells. (**F**) IHC staining of sequential section of panel **E**. (**G**) Type II pneumocyte hyperplasia is visible on 6 dpi, as highlighted further in inset. (**H**) IHC staining of sequential section of panel **G** indicates viral antigen has been mostly cleared from remodeling tissue. (**I**) On 6 dpi, fibrin is consolidating into hyaline membranes (arrows). (**J**) IHC staining of sequential section of panel **I**. Magnification: **A**, **B**, **C** and **D** 4×; **E**, **F**, **G**, **H**, **I** and **J** 40×; inset in panel **E** and **G** 100×.

**Figure 5:**
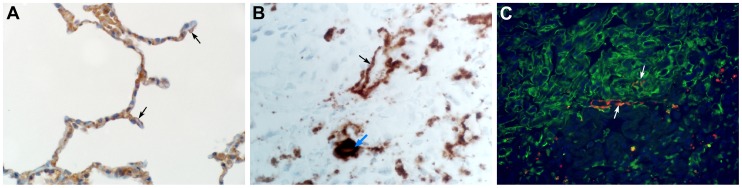
The cellular tropism of MERS-CoV. (**A**) DPP4 is evident on type I pneumocytes (arrow), bronchiolar epithelial cells and smooth muscle cells. (**B**) *In situ* hybridization demonstrating the presence of viral RNA in type I pneumocytes (black arrow) and alveolar macrophages (blue arrow). (C) Type I pneumocytes (green) are lost following infection with MERS-CoV (lower right). Viral antigen (red) can be observed in type I pneumocytes (arrow). Magnification: **A** 500×; **B** 200×; **C** 500×.

Lungs from marmosets necropsied at 3 and 4 dpi all showed multifocal to coalescing, moderate to marked acute bronchointerstitial pneumonia (Fig. 4A,C). The pneumonia tended to be centered on small caliber and terminal bronchioles and extended into the adjacent pulmonary parenchyma. Viral antigen was exclusively associated and located throughout regions that contained pathological changes (Fig. 4B,D,F,H,J). The bronchiolar epithelium was frequently eroded, leaving attenuated bronchiolar epithelial cells. Affected bronchioles were filled with small to moderate numbers of macrophages and neutrophils, and occasionally small amounts of fibrin and edema (Fig. 4E,G). The adjacent alveolar interstitium was thickened with congestion, edema and fibrin and moderate numbers of macrophages and neutrophils (Fig. 4E, Table S4). Alveolar spaces contained moderate to marked numbers of pulmonary macrophages and neutrophils; multifocally there was pulmonary edema, fibrin, and less frequently hemorrhage (Fig. 4E). There were also rare multinucleate syncytia within alveolar spaces (Fig. 4E). At 6 dpi multifocal to coalescing areas of acute pneumonia were still visible; however, there were also extensive areas of type II pneumocyte hyperplasia (Fig. 4G) and consolidation of pulmonary fibrin resulting in multifocal hyaline membranes (Fig. 4I). These changes are consistent with a transition from acute to a more chronic reparative stage of pneumonia. Regions that were undergoing tissue remodeling showed evidence of clearing of viral antigen (Fig. 4 H). The distribution of DPP4 in marmoset lungs included type I pneumocytes (Fig. 5A) as well as bronchiolar epithelial cells and smooth muscle cells. Consistent with this location of DPP4, a two-color fluorescent staining for cytokeratin and viral antigen, as well as *in situ* hybridization to detect viral RNA, identified type I pneumocytes and alveolar macrophages as the primary cell type for MERS- CoV replication (Fig. 5 B,C). One of the surviving animals (CM7) had to be euthanized prior to the scheduled end of the study (48 dpi) and was found to have severe aspiration pneumonia. The lungs from the other surviving animal (CM8) appeared normal at necropsy 55 dpi. Viral antigen was not detected by IHC in either animal indicating that these animals had resolved MERS-CoV infection. All other lesions noted in the remaining tissues, such as interstitial nephritis in the kidney and the presence of giant cells in the adrenal gland, consistent with extramedullary hematopoiesis, were not considered to be clinically significant since they are typical, incidental findings in common marmosets [22].

### Viral RNA is present in swabs and blood

**Figure 6:**
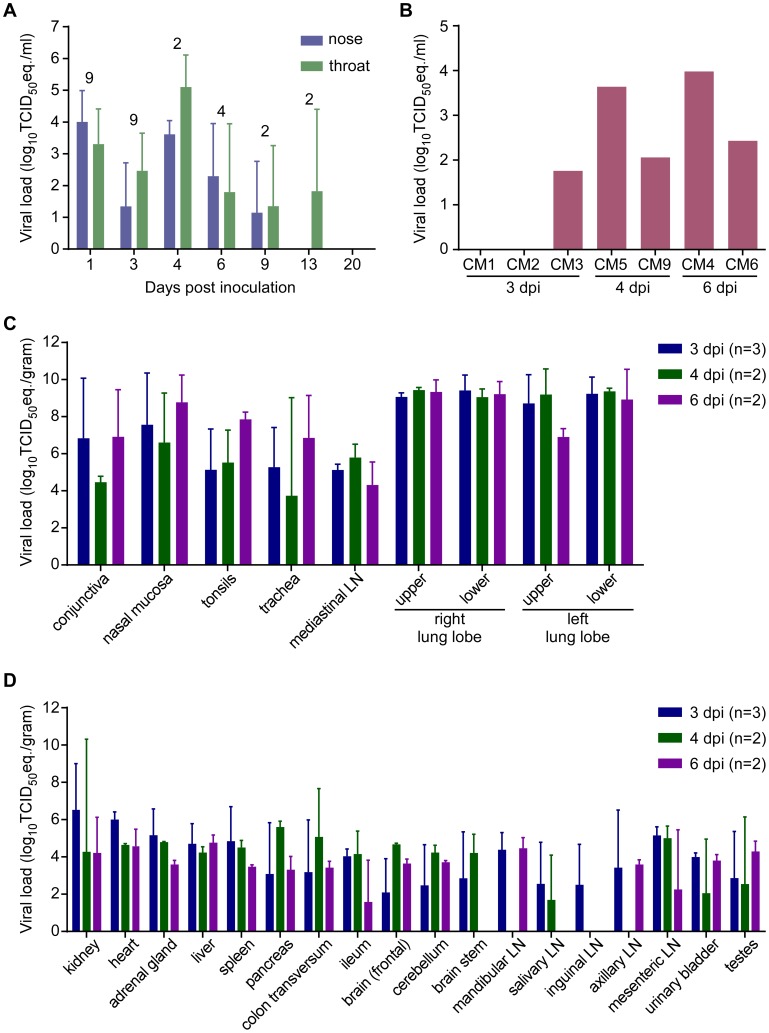
Viral load from marmosets inoculated with MERS-CoV in nasal and throat swabs (A), blood (B) and tissues (C,D). Nasal and throat swabs were collected at exams while blood samples were only collected at necropsy. RNA was extracted and viral load was determined as TCID_50_ equivalents by qRT-PCR. The number of animals included in the analysis at each time point in (A) is indicated above the graph. Respiratory (**C**) and other tissues (**D**) were collected at necropsy on the indicated days post-inoculation. RNA was extracted and viral load determined as TCID_50_ equivalents per gram of tissue by qRT-PCR.

On 1, 3, 6, 9, 13 and 20 dpi, nasal and oropharyngeal swabs were obtained from all remaining animals; on 4 dpi before euthanasia, swabs were also obtained from CM5 and CM9. All but one of the nasal swabs collected on 1 dpi were positive for the presence of viral RNA by qRT-PCR (Fig. 6A). By 3 dpi, viral loads in nasal and oropharyngeal swabs were lower than on 1 dpi. However, swabs collected from CM5 and CM9 at the time of euthanasia at 4 dpi contained the highest viral loads. In the surviving animals, viral loads in the swabs decreased over time and all swabs were negative by 20 dpi (Fig. 6A). A terminal blood sample was collected from animals CM1–CM6 and CM9. CM5, euthanized on 4 dpi, and CM4, euthanized on 6 dpi, were viremic (Fig. 6B), although viral loads in these blood samples were very low and virus isolation attempts on blood samples were not successful (data not shown).

### Marmosets develop disseminated viral infection

Upon necropsy of the animals on 3, 4 and 6 dpi, tissue samples were collected and subsequently analyzed for the presence of viral RNA by qRT-PCR. High viral loads were detected mainly in respiratory tissues (Fig. 6C). Viral loads in lung tissue samples obtained from the animals on 3 dpi were very high, reaching up to 10^7^ TCID_50_ eq./gram. Viral loads in the lungs did not decrease between 3 and 6 dpi. Viral RNA was detected in all tested tissues, albeit not in every animal (Fig. 6D). Viral RNA was not detected in the lungs, or other tissues, from the two surviving animals that were euthanized on 48 and 55 dpi; however, both of these animals seroconverted indicating that they had been exposed to MERS-CoV. Virus isolation attempts were performed on nasal mucosa, trachea, lung and kidney samples. In all animals except CM6 (6 dpi), virus could be isolated from one or more lung lobes (Table S5). Virus was isolated from all trachea samples collected on 4 and 6 dpi. Virus was not isolated from any of the kidney samples (Table 5).

### Transcriptional changes support increased inflammatory processes

To characterize transcriptional changes as a result of MERS-CoV infection, we performed whole transcriptional sequencing on RNA samples extracted from the right lower lobe of marmoset lungs collected at necropsy. Samples were collected from areas of the lung that contained gross lesions. These were compared to the total RNAseq data from the lung of a single uninfected marmoset obtained through the Non-Human Primate Reference Transcriptome Resource (http://www.nhprtr.org) [23]. We reasoned that molecules induced by infection that play a role in respiratory pathogenesis would at least double in abundance over the course of infection. Therefore we identified mean differentially expressed (DE) transcripts at each time point with at least 2-fold change compared to the uninfected control.

**Figure 7:**
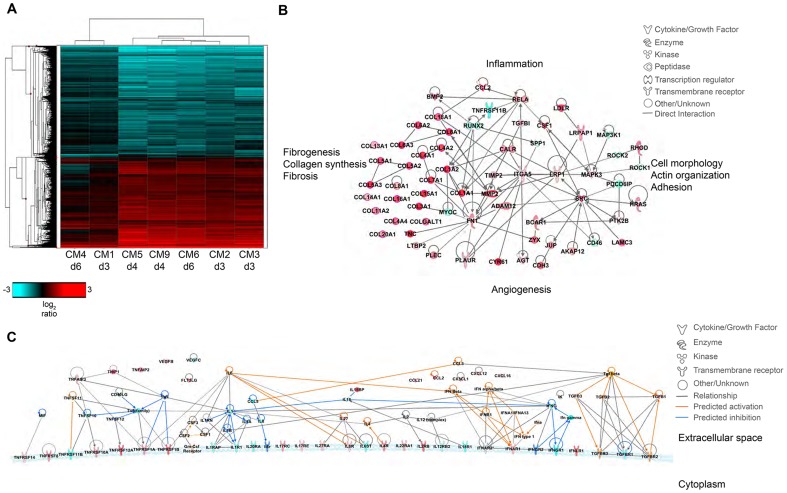
Transcriptional signatures of MERS-CoV infection in marmoset lungs. (**A**) 2-dimensional hierarchal clustering of DE transcripts from infected lungs. The heatmap shows log2 expression of transcripts with greater than 2-fold change relative to uninfected marmoset lung among all the MERS-CoV-infected samples. The row dendrogram shows clusters of gene expression and the column dendrogram shows clusters of samples with similar transcriptional profiles. (**B**) Functional network of genes demonstrating the functional relationship between inflammatory mediators and connective tissue adhesions. (**C**) Molecule activity prediction (MAP) network of cytokines, chemokines, growth factors, and receptors at 4 dpi. Red molecules are those upregulated in the data set, teal molecules are those downregulated in the dataset, orange molecules are those predicted to be activated, and dark blue molecules are those predicted to be inhibited. Orange lines indicate a predicted activation effect, blue lines indicate a predicted inhibitory effect, and gray lines indicate a relationship without a known or predicted activity or inhibition. dpi. Red molecules are those upregulated in the data set, teal molecules are those downregulated in the dataset, orange molecules are those predicted to be activated, and dark blue molecules are those predicted to be inhibited. Orange lines indicate a predicted activation effect, blue lines indicate a predicted inhibitory effect, and gray lines indicate a relationship without a known or predicted activity or inhibition.

Molecular profiles were functionally similar over the course of sampling (Fig. 7A); however, there were some alterations in functional category enrichment and magnitude of expression relative to the uninfected control. Throughout infection, pathways associated with chemotaxis and cell migration, cell cycle progression, cell proliferation, and fibrogenesis were increasingly activated relative to the uninfected control lung, with higher expression and greater pathway enrichment occurring at 4 and 6 dpi than at 3 dpi. We also observed activation of pathways associated with inflammation, vascularization, endothelial activation, proliferation of smooth muscle cells, and tissue repair, indicating that MERS-CoV infection induces tissue differentiation in marmosets consistent with development of pulmonary fibrosis (Fig. 7B, Table S6).

Despite the observed severe lung pathology, robust antiviral transcriptional responses were induced in the marmoset lungs. Up regulation of innate immune genes such as pattern recognition receptors, interferon-stimulated genes, inflammatory cytokines and signaling molecules, as well as adaptive immune responses such as lymphocyte signaling, proliferation, and differentiation, immunoglobulin production, antigen presentation, and T cell costimulation were observed. We were able to detect many common serum cytokine transcripts using RNAseq, which unfortunately cannot be measured in immunoassays as these are not available for common marmosets. Notably, we detected down regulation of interferon gamma (IFNγ) and its receptor (IFNGR1) at multiple time points, and no expression of interferon beta (IFNβ), consistent with reports that MERS-CoV infection attenuates these cytokines [24]–[29]. We did observe up regulation of the type I interferon receptor (IFNAR1, IFNAR2) and interferon stimulated genes (ISGs) induced by type I interferon responses, in addition to the type III interferon receptor (IFNLR1), (Table S7). We also observed significant down regulation of IL-8 and IL-18 at all time points, and up regulation of IL-27 for all animals. IL-1β was up regulated in the 4 dpi samples, but down regulated at 3 and 6 dpi. As cytokine transcripts generally have a short half-life, in some cases we were not able to detect DE cytokine transcripts, but were able to detect changes in their receptors or molecules associated with cytokine signaling. We observed up regulation of the receptors for IL-2 (IL2RB), IL-4 (IL4R), IL-6 (IL-6R), IL-17 (IL17RC, IL17RE), IL-22 (IL22RA1), and IL-27 (IL27RA), and down regulation of receptors for IL-1 (IL1R1, IL1RAP), IL-12 (IL12RB2), IL-18 (IL18R1), and IL-20 (IL20RA) (Table S7). Using the Molecule Activity Prediction (MAP) tool in IPA, we were able to generate a network of these molecules showing the predicted activity of cytokines not identified in the dataset at 4 dpi (Fig. 7C). The MAP tool utilizes relationships with molecules neighboring the DE transcripts in this dataset to predict transcriptional behavior. These relationships are based on published work curated in the IPA knowledgebase, and provide insight into the dynamics of all molecules within a given network or pathway generated using the primary transcriptional data from this experiment. While type I interferons, IL-2, IL-4, and IL-6 were predicted to be induced, type II interferons and the pro-inflammatory cytokines IL-1 and TNFα were predicted to be at least partially inhibited. While transcriptional induction of proinflammatory cytokines such as IL-1 and TNFα was expected, especially given the extent of inflammation in the lungs, the time points for sampling may have been too late to catch TNFα induction as it is one of the first cytokines induced during inflammation. Its induction is supported by downstream effectors such as NFκB, RelA, and RelB and many accessory proteins that were unregulated at the time points investigated. Moreover, as TNFα signaling tends to be down regulated rapidly, the up regulation of SOCS3, which is induced by TNFα as part of a negative feedback loop that ultimately results in suppression of TNFα and IL-1, was observed at all time points.

## Discussion

In MERS-CoV-infected common marmosets, clinical disease was more severe than in the rhesus macaque, was of longer duration and resulted in euthanasia of some animals. Viral loads in the lungs were up to 1000 times higher than those in the rhesus macaque lungs (mean viral load in the lungs 1.2×10^3^ in rhesus vs. 1.5×10^6^ in marmosets on 3 dpi; 6.8×10^2^ in rhesus vs. 2.1×10^6^ in marmosets on 6 dpi) [15]. Two of the six animals that were not euthanized at the scheduled 3 dpi necropsy had to be euthanized due to severity of disease, making this the first lethal MERS-CoV animal model. In both the marmoset and rhesus macaque [15] models, viral replication occurred predominantly in the lower respiratory tract; however, in marmosets MERS-CoV RNA was also detected in the blood. This is suggestive of a more systemic dissemination that was corroborated by the detection of viral RNA in nearly all tested tissues in all infected animals. Taken together, the data from the common marmoset model suggest that this model more closely recapitulates severe, even lethal, human disease caused by MERS-CoV. This differs from the rhesus macaque model for MERS-CoV [13]–[15], which more closely resembles mild to moderate human disease.

While viral RNA was detected in kidney samples from five out of seven marmosets, no histological abnormalities were observed, virus could not be isolated and viral antigen was not detected by immunohistochemistry. Elevations in creatinine and blood urea nitrogen levels were noted on 4 dpi in the two animals that were euthanized, suggesting some degree of kidney involvement during infection; however, the lack of evidence of virus replication in this tissue suggests that this is not a direct viral effect. The acute renal failure in some patients [21] may be a secondary effect of ARDS [30] or other comorbidities and not primarily the result of direct MERS-CoV damage to the kidneys. While evidence of pulmonary fibrosis was not yet observed histologically at the time points investigated, transcriptional evidence of the onset of fibrosis was extensive. Previous studies of severe acute respiratory syndrome coronavirus (SARS-CoV) have demonstrated that pulmonary fibrosis was a major mechanism of disease progression, and that lung inflammation caused by infection induced fibrogenic transcriptional programs [31]–[33]. In the marmosets, the role of fibrosis is unknown and needs to be further clarified and supported by data from human cases. We anticipate that studies using larger groups of marmosets, additional control animals, and samples collected at later time points post-infection will confirm the histologic course of fibrosis progression, as well as the transcriptional events underlying these pathogenic processes.

Currently, the only small animal MERS-CoV challenge model available, requires transduction of animals with an adenovirus vector expressing human DPP4 [12]. Although this is a very useful model, MERS-CoV infection in this model is highly dependent on the transduction of cells and level of DPP4 expression from the adenovirus vector and thus does not necessarily reflect the natural disease process. Therefore, therapeutics indicated to inhibit MERS-CoV in *in vitro* studies likely need to be tested in one of the two described nonhuman primate models. As such, marmosets should likely serve as the animal of choice for future therapeutic studies where possible. Not only does the more severe, and potentially lethal disease set a higher bar for protection, it would also allow a greater differentiation to be made between disease in untreated animals versus treated animals, currently a limitation of the rhesus macaque model [7]. The marmoset model also allows the evaluation of intervention strategies at later time points as the disease process in the rhesus model is rapid and quite transient. However, late treatment that targets the virus, as with many countermeasures, is unlikely to be successful once significant lung damage has already occurred as was observed by the lack of success of very late treatment with ribavirin and interferon in human MERS-CoV cases [34]. To enable treatment of patients prior to severe lung injury, future transcriptional studies may yield early indicators of disease progression that can be used as diagnostic or prognostic tests to improve clinical management. The development of the more severe marmoset model will ensure a better pre-clinical analysis of treatments prior to proceeding to clinical trials in humans. As such, this new MERS-CoV disease model is a significant contribution to reducing the impact of MERS-CoV on global public health.

## Materials and Methods

### Ethics statement

All animal experiments were approved by the Institutional Animal Care and Use Committee (IACUC) of the Rocky Mountain Laboratories (RML), and performed following the guidelines of the Association for Assessment and Accreditation of Laboratory Animal Care, International (AAALAC) by certified staff in an AAALAC-approved facility, following the guidelines and basic principles in the United States Public Health Service Policy on Humane Care and Use of Laboratory Animals and the Guide for the Care and Use of Laboratory Animals. All procedures were carried out under a combination of Ketamine and isoflurane anesthesia by trained personnel under the supervision of veterinary staff and all efforts were made to provide for the welfare and to minimize any suffering in accordance with the “Weatherall report for the use of non-human primates”. Animals were housed in adjoining individual primate cages allowing social interactions, under controlled conditions of humidity, temperature and light (12-hour light/12-hour dark cycles). Food and water were available *ad libitum*. Animals were monitored twice daily (pre- and post-challenge) and fed a combination of commercial New World monkey chow, rice cereal supplemented with calcium, ZuPreem marmoset, wax worms/larvae and fruit twice daily by trained personnel. Endpoint criteria, as specified by the RML IACUC approved score parameters, were used to determine when animals should be humanely euthanized. Animals were euthanized by exsanguination under deep isoflurane anesthesia. The work with infectious HCoV-EMC/2012 was approved under BSL3 conditions by the Institutional Biosafety Committee (IBC). Sample inactivation was performed according to standard operating procedures approved by the IBC for removal of specimens from high containment.

### Study design

In order to establish a more severe animal model for MERS-CoV, the interaction of the common marmoset DPP4 with the MERS-CoV spike protein was modeled. Subsequently, experimental inoculation of common marmosets was performed to determine whether they would serve as an improved disease model. Nine male common marmosets (*Callithrix jacchus*; 2–6 years old) were randomly assigned a number (CM1–CM9) and subsequently inoculated with MERS-CoV (strain HCoV-EMC/2012) intranasally with 100 µl in each nare, 500 µl orally, 500 µl intratracheally and 50 µl in each eye with DMEM containing 4×10^6^ TCID_50_/ml (total dose 5.2×10^6^ TCID_50_). Necropsies of three animals were scheduled on 3 dpi (CM1–CM3) and 6 dpi (CM4-6). The three remaining animals (CM7–CM9) were not scheduled for euthanasia, but were used to study survival and seroconversion upon inoculation of animals with MERS-CoV (Fig. 2A). The animals were observed twice daily for clinical signs of disease and scored using a clinical scoring system prepared for common marmosets (Table S2). The in-study euthanasia criteria were established prior to the start of the experiment based on the scoring sheet and euthanasia was indicated at a clinical score of 35 or above (Table S2). During the course of the study, animals CM5 and CM9 were euthanized on 4 dpi as they reached euthanasia criteria. On 1, 3, 6, 9, 13 and 20 days post inoculation, clinical exams were performed on anaesthetized animals, x-rays were taken and nasal and oral swabs were collected in 1 ml DMEM with 50 U/ml penicillin and 50 µg/ml streptomycin. Temperature was monitored with IPTT-300 temperature probes (BMDS) that were injected interscapularly prior to the start of the experiment. Blood was collected prior to the start of the study and at euthanasia for hematology and blood chemistry analysis. The total white blood cell count, lymphocyte, platelet, reticulocyte, and red blood cell counts, hemoglobin, hematocrit values, mean cell volume, mean corpuscular volume, and mean corpuscular hemoglobin concentrations were determined from EDTA blood with the HemaVet 950FS+ laser-based hematology analyzer (Drew Scientific). Samples of the following tissues were collected: conjunctiva, nasal mucosa, tonsil, mandibular lymph node, salivary gland, trachea, all four lung lobes, mediastinal lymph node, inguinal lymph node, axillary lymph node, mesenteric lymph node heart, liver, spleen, kidney, adrenal gland, pancreas, ileum, colon transversum, urinary bladder, testes, frontal brain, cerebellum and brain stem.

### Amino acid and binding energy comparison between human and marmoset DPP4

Amino acid sequence alignment was generated using the human, ferret and marmoset DPP4s (accession numbers NP_001926.2, DQ266376 and XM_002749392 respectively) using CLUSTALW2 [35]. The human DPP4 structure model (PDB ID: 4KR0, Chain A) was used as template to highlight the location of the amino acid differences between the human and marmoset DPP4s.The initial model was built using Nest [36] based on the amino acid alignment and the human DPP4 structure. The resulting structural model was briefly optimized using the TINKER minimization program “minimize.x” with OPLS all-atom force field and L-BFGS quasi-Newton optimization algorithm [37]. For the marmoset and ferret DPP4, the RBD/DPP4 complex model was generated by merging the RBD domain (PDB ID: 4KR0, Chain B) with the DPP4 model, which was then subjected to the binding energy calculation using an all-atom distance-dependent pairwise statistical potential, DFIRE [38]. The energy difference between the complex and two individual structures - DPP4 and RBD - was taken as the binding energy.

### Virus and cells

HCoV-EMC/2012 [21] was kindly provided by the Department of Viroscience, Erasmus Medical Center, Rotterdam, The Netherlands and propagated once in VeroE6 cells in DMEM (Sigma) supplemented with 2% fetal calf serum (Logan), 1 mM L-glutamine (Lonza), 50 U/ml penicillin and 50 µg/ml streptomycin (Gibco) (virus isolation medium). VeroE6 and LLC-MK2 cells were maintained in DMEM supplemented with 10% fetal calf serum, 1 mM L-glutamine, 50 U/ml penicillin and 50 µg/ml streptomycin.

### Histopathology and immunohistochemistry

Histopathology and immunohistochemistry were performed on marmoset tissues. After fixation for 7 days in 10% neutral-buffered formalin and embedding in paraffin, tissue sections were stained with hematoxylin and eosin (HE). To detect HCoV-EMC/2012 antigen, immunohistochemistry was performed using a rabbit polyclonal antiserum against HCoV-EMC/2012 (1∶1000) as a primary antibody for detection of HCoV-EMC/2012 antigen. Immunohistochemistry for DPP4 (CD26) was performed with a rabbit polyclonal antiserum (1∶400) (Abcam). To confirm the cell type of infected cells a subset of specimens were stained with an antibody against cytokeratin (1∶100) (Dako) and the rabbit polyclonal against HCoV-EMC/2012. Fluorescently labeled goat anti-mouse (AlexaFluor 488) goat anti-rabbit (AlexaFluor 594) were used for detection. Tissues from an uninfected control animal was obtained from Primate Biologicals and used to validate all *in situ* and immunohistochemistry procedures.

### RNA extraction

RNA was extracted from swab and whole blood samples using the QiaAmp Viral RNA kit (Qiagen) according to the manufacturer's instructions. Tissues (30 mg) were homogenized in RLT buffer and RNA was extracted using the RNeasy kit (Qiagen) according to the manufacturer's instructions. For whole transcriptome sequencing, tissues collected from the right lower lobe of the lung were homogenized in TRIzol reagent and frozen at −80°C. The TRIzol was phase separated and total RNA was further purified from the aqueous phase using the miRNeasy kit (Qiagen) according to the manufacturer's instructions.

### Virus isolation

Weighed tissue samples were homogenized in a TissueLyzer II (Qiagen) after addition of 1 ml DMEM. Homogenates were centrifuged to pellet cellular debris and 10-fold dilutions of homogenate were made and subsequently inoculated onto VeroE6 and LLC-MK2 cells for virus isolation. After 1 hr, cells were washed once with DMEM and supplemented with virus isolation medium. Cells were scored for cytopathic effect 5 days following infection.

### Quantitative PCR

For detection of viral RNA, 5 µl RNA was used in a one-step real-time RT-PCR upE assay [39] using the Rotor-Gene probe kit (Qiagen) according to instructions of the manufacturer. In each run, standard dilutions of a titered virus stock were run in parallel, to calculate TCID_50_ equivalents in the samples.

### Total RNA library preparation

Total RNA libraries were constructed using the Illumina TruSeq Stranded Total RNA Preparation Kit (Illumina) according to the manufacturer's guide. Input RNA was rRNA reduced using the Ribo-Zero Magnetic Kit (Human/Mouse/Rat), (Epicentre), and rRNA reduction was verified by BioAnalyzer 2100 (Agilent Technologies). Libraries were quality controlled and quantitated using the BioAnalzyer 2100 system and mass measurement using QuBit (Life Technologies). The libraries were clonally amplified on a cluster generation station using Illumina version 2 MiSeq reagents to achieve a target density of approximately 1000 K–1200 K clusters/mm2 on the flow cell. The resulting libraries were then sequenced on an Illumina MiSeq system at single reads of 50 bp, and were processed on the system to generate quality control metrics and FASTQ files with MiSeq Reporter 2.3.32.

### Next generation sequencing

Raw reads were trimmed to 50 base pairs (bp) and adapter sequences were removed. We mapped the 50 bp reads to a custom-compiled set of known ribosomal sequences (human, mouse) using the short-read aligner software Bowtie v. 1.0.0 [40] to remove potential rRNA sequences. The remaining unmapped reads corresponding to MERS-CoV genome (GenBank accession no. JX869059.2) were also removed using Bowtie. All the remaining reads were mapped to the common marmoset (*Callithrix jacchus*) genome assembly (Genome assembly: C_jacchus3.2.1, GCA_000004665.1) downloaded from Ensembl (version 74) using STAR v.2.3.0.1 [41]. Gene level quantification was obtained using HT-seq (http://www-huber.embl.de/users/anders/HTSeq/doc/overview.html) with Ensembl annotation (v74). For the downstream analyses we only retained those transcripts with at least 40 raw read counts in at least one sample. The raw read counts were normalized using edgeR [42]. For the calculation of expression ratios, an offset of 5 was added to the normalized expression levels, i.e. counts per million (cpm) values to dump down variations due to low abundances. We identified transcripts with greater than 2-fold change relative to the uninfected control, and used Spotfire DecisionSite (Tibco) to perform hierarchical clustering using the unweighted pair group method with arithmetic means (UPGMA) method, and generate heatmaps. Functional enrichment analysis was performed using Ingenuity Pathway Analysis (IPA). Raw data were deposited into the Sequence Read Archive (SRA) via the Gene Expression Omnibus (accession number pending).

### Data and materials availability

GEO Accession #GSE55023.

## Supporting Information

Figure S1 Temperature, hematology and clinical blood chemistry from common marmosets inoculated with MERS-CoV. (**A**) Temperature was monitored via injectable temperature probes. Hematology values for (**B**) white blood cell, (**C**) neutrophils and (**D**) red blood cells were determined on a HemaVet. Clinical blood chemistry values for the liver function (**E**) aspartate aminotransferase (AST), (**F**) alanine aminotransferase (ALT), (**G**) alkaline phosphatase (ALP), (**H**) bilirubin; kidney function (**I**) blood urea nitrogen (BUN), (**J**) creatinine; and (**K**) total protein and (**L**) albumin were determined.
(TIF)

Figure S2 Gross lung parameters at necropsy from common marmosets inoculated with MERS-CoV. (**A**) Gross pathology scores representing the area of lesion on dorsal and ventral surface of the upper and lower lung lobes. Shaded area represents the range of values, solid line indicates the median value, error bar represents the 95% confidence interval. P values from 2-way ANOVA are indicated above the graph. (**B**) Lung weight to body weight ratio.
(TIF)

Table S1 Clinical observations in common marmosets inoculated with MERS-CoV between 1 and 6 dpi. Animals were observed twice daily and clinical parameters were recorded.
(DOCX)

Table S2 Clinical score sheet for common marmosets inoculated with MERS-CoV.
(DOCX)

Table S3 Summary of radiographic changes of common marmosets inoculated with MERS-CoV. The quality and location of interstitial infiltration observed from ventral-dorsal and lateral x-rays is indicated.
(DOCX)

Table S4 Histopathology score in MERS-CoV inoculated common marmosets based on area of tissues affected by microscopic lesions.
(DOCX)

Table S5 Virus isolation from tissues of common marmosets inoculated with MERS-CoV in VeroE6 or LLC-MK2 cells.
(DOCX)

Table S6 Differentially expressed genes identified to be involved in fibrosis pathways.
(DOCX)

Table S7 Differentially expressed genes identified to be involved in antiviral pathways.
(DOCX)
