# Correction: Infection with MERS-CoV Causes Lethal Pneumonia in the Common Marmoset

**DOI:** 10.1371/journal.ppat.1004431

**Published:** 2014-09-09

**Authors:** The PLOS Pathogens Staff

The incorrect version of Figure 6 is published in the article. Please see the correct version of Figure 6 here, with the proper viral loads.

**Figure 6:**
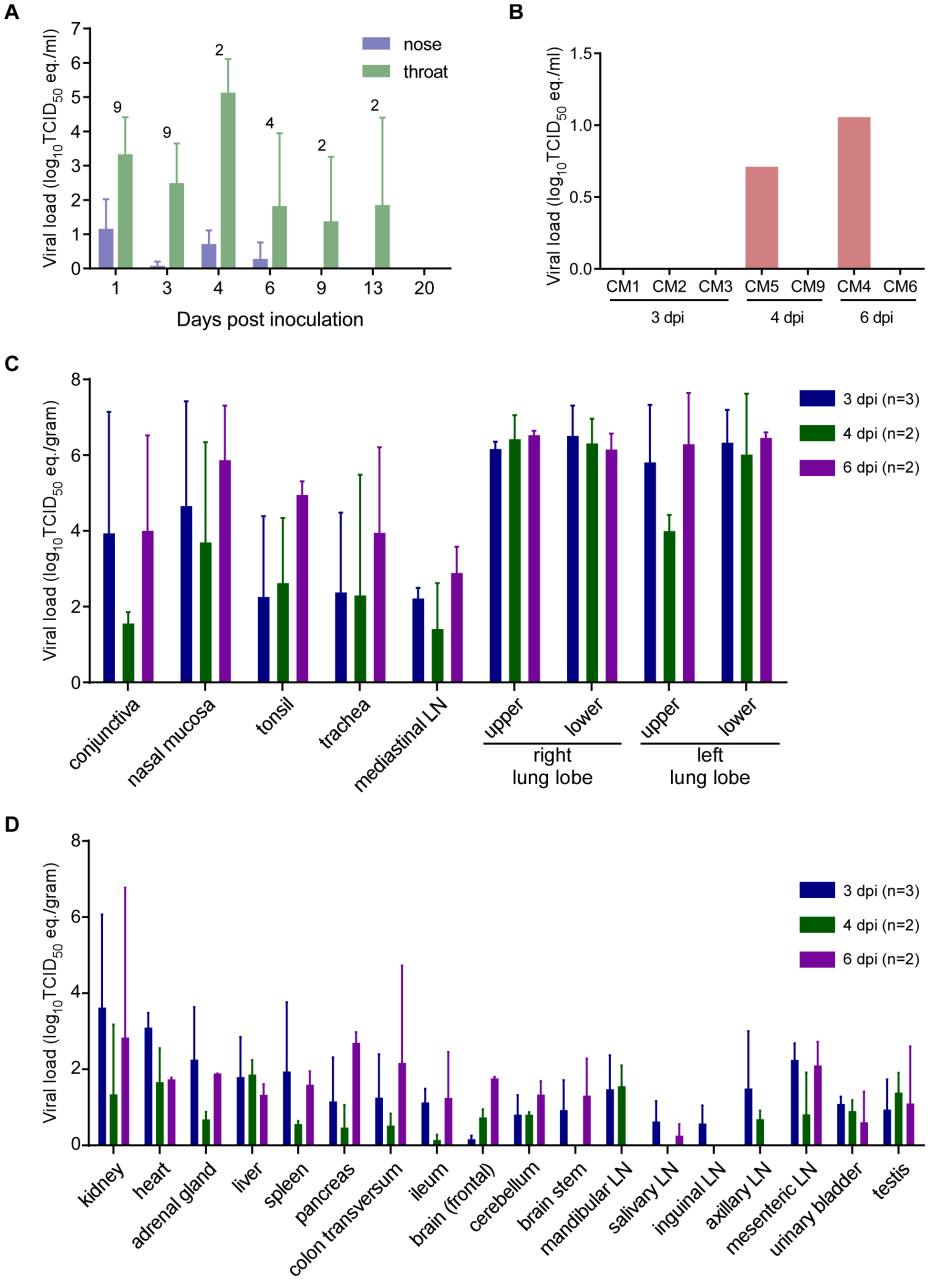
Viral load from marmosets inoculated with MERS-CoV in nasal and throat swabs (A), blood (B) and tissues (C,D). Nasal and throat swabs were collected at exams while blood samples were only collected at necropsy. RNA was extracted and viral load was determined as TCID_50_ equivalents by qRT-PCR. The number of animals included in the analysis at each time point in (A) is indicated above the graph. Respiratory (**C**) and other tissues (**D**) were collected at necropsy on the indicated days post-inoculation. RNA was extracted and viral load determined as TCID_50_ equivalents per gram of tissue by qRT-PCR.
