# Supplementary figures and images for: Infection with MERS-CoV Causes Lethal Pneumonia in the Common Marmoset
Source: PLoS Pathog. 2014 Aug 21;10(8):e1004250. doi: 10.1371/journal.ppat.1004250 (PMC4140844; doi:10.1371/journal.ppat.1004250)

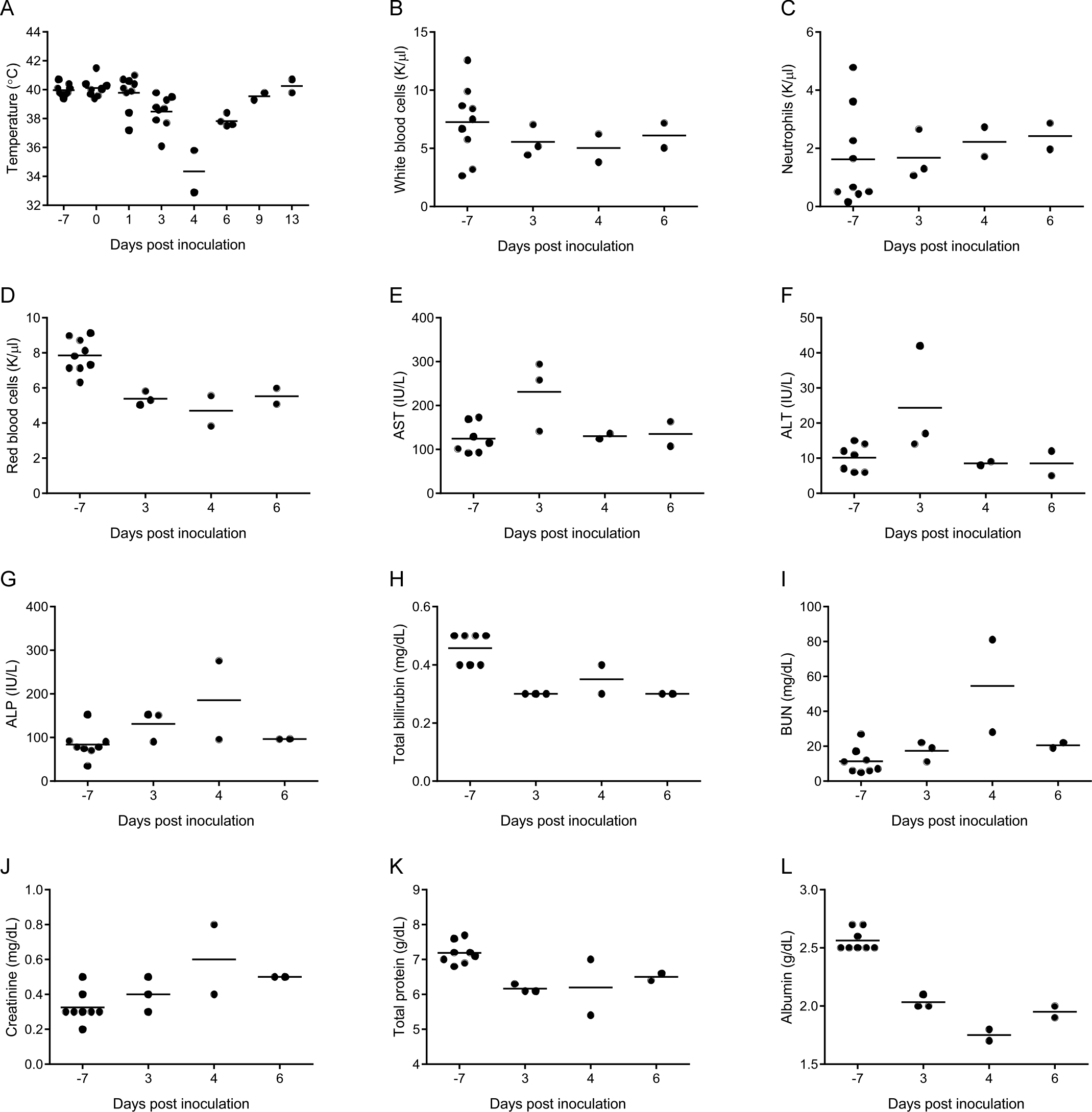

Supplement: Figure S1 — Temperature, hematology and clinical blood chemistry from common marmosets inoculated with MERS-CoV. (A) Temperature was monitored via injectable temperature probes. Hematology values for (B) white blood cell, (C) neutrophils and (D) red blood cells were determined on a HemaVet. Clinical blood chemistry values for the liver function (E) aspartate aminotransferase (AST), (F) alanine aminotransferase (ALT), (G) alkaline phosphatase (ALP), (H) bilirubin; kidney function (I) blood urea nitrogen (BUN), (J) creatinine; and (K) total protein and (L) albumin were determined. (TIF) [file ppat.1004250.s001.tif]

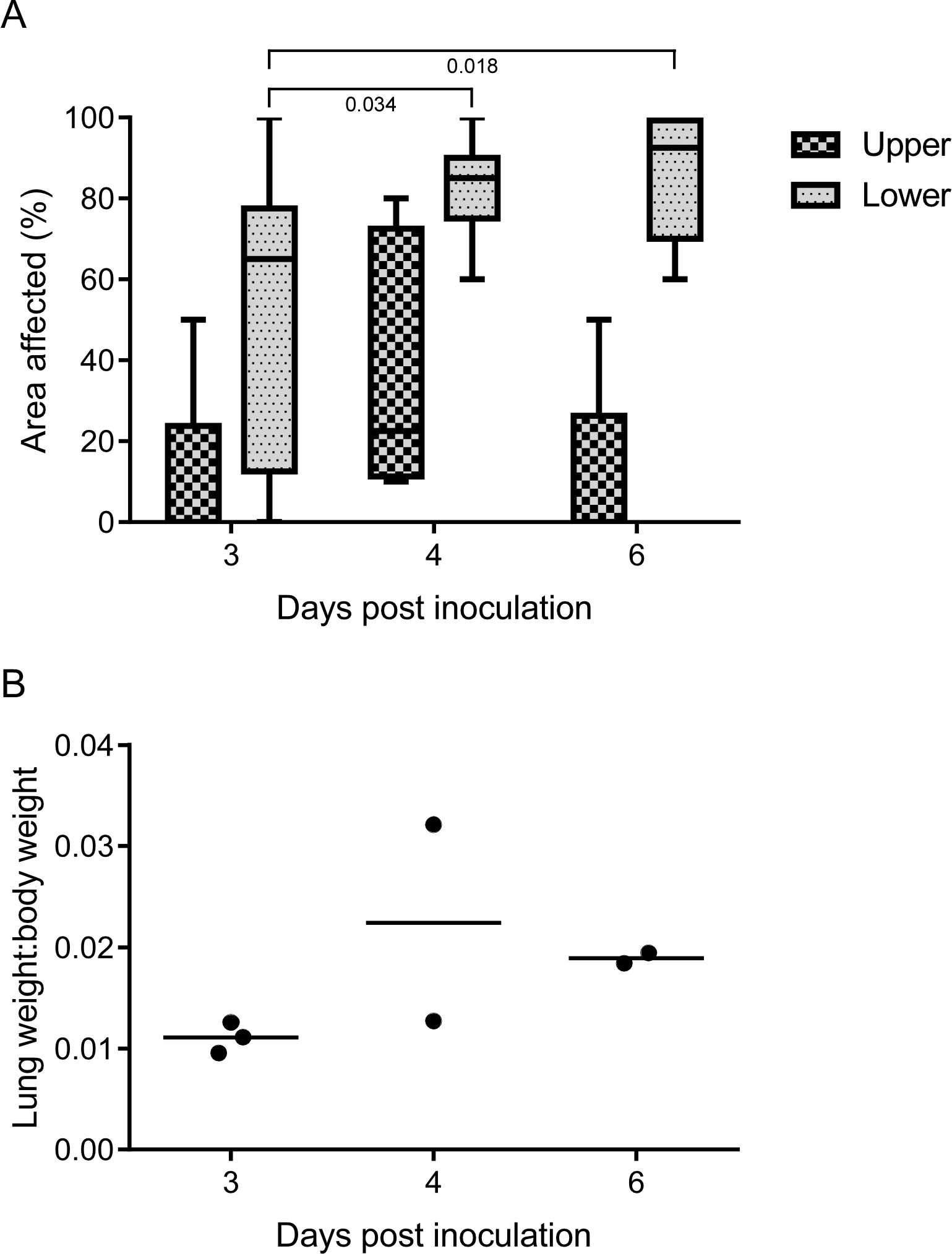

Supplement: Figure S2 — Gross lung parameters at necropsy from common marmosets inoculated with MERS-CoV. (A) Gross pathology scores representing the area of lesion on dorsal and ventral surface of the upper and lower lung lobes. Shaded area represents the range of values, solid line indicates the median value, error bar represents the 95% confidence interval. P values from 2-way ANOVA are indicated above the graph. (B) Lung weight to body weight ratio. (TIF) [file ppat.1004250.s002.tif]
