# Supplementary material for: Infection with MERS-CoV Causes Lethal Pneumonia in the Common Marmoset
Source: PLoS Pathog. 2014 Aug 21;10(8):e1004250. doi: 10.1371/journal.ppat.1004250 (PMC4140844; doi:10.1371/journal.ppat.1004250)
Supplement: Table S1 — Clinical observations in common marmosets inoculated with MERS-CoV between 1 and 6 dpi. Animals were observed twice daily and clinical parameters were recorded. (DOCX) [file ppat.1004250.s003.docx]

**Table S1.** Clinical observations in common marmosets inoculated with MERS-CoV between 1 and 6 dpi. Animals were observed twice daily and clinical parameters were recorded.

|  | **Day 1** | **Day 2** | **Day 3** | **Day 4** | **Day 5** | **Day 6** |
| --- | --- | --- | --- | --- | --- | --- |
| **CM1** | resp. ↑^1^ | less active; loss of appetite | resp. ↑; less active; loss of appetite; hunched | N/A^2^ | N/A | N/A |
| **CM2** | resp. ↑↑^3^ | resp.↑↑; less active; loss of appetite; hunched | resp. ↑↑; less active; loss of appetite; hunched | N/A | N/A | N/A |
| **CM3** | -^4^ | resp. ↑; less active; loss of appetite | resp. ↑↑; less active; loss of appetite; hunched;  needs more prompting to move | N/A | N/A | N/A |
| **CM4** | - | resp. ↑; less active; loss of appetite; hunched | resp. ↑; less active; loss of appetite; hunched | resp. ↑; less active; loss of appetite; hunched; needs more prompting to move | resp. ↑↑; less active; loss of appetite; hunched; needs more prompting to move; labored breathing; very pale appearance | resp. ↑↑; less active; hunched; needs more prompting; labored breathing; very pale appearance; anorexic; dark mucus membranes; dehydrated |
| **CM5** | resp. ↑ | resp. ↑; less active; hunched; needs more prompting to move;  anorexic | resp. ↑↑; less active; hunched; needs  more prompting to move; labored breathing; anorexic | resp. ↑↑; almost impossible to prompt to move; little response to human presence; anorexic; dehydrated; bloody oral discharge; mildly cyanotic | N/A | N/A |
| **CM6** | resp. ↑; piloerection | resp. ↑; loss of appetite | resp. ↑↑; loss of appetite; open mouth breathing; pale appearance | resp. ↑↑; less active; loss of appetite; hunched; open mouth breathing; pale appearance | resp. ↑↑; less active; hunched; anorexic; labored breathing; pale appearance | resp. ↑↑; less active; hunched;  anorexic; labored breathing; pale/blue appearance |
| **CM7** | resp. ↑ | resp. ↑; less active; loss of appetite | resp. ↑; less active; loss of appetite | resp. ↑; less active; loss of appetite | resp. ↑; less active; hunched; anorexic; pale appearance | resp. ↑↑; less active; hunched; anorexic |
| **CM8** | - | resp. ↑; less active; loss of appetite;  hunched | resp. ↑; less active; loss of appetite;  hunched | resp. ↑; less active; loss of appetite;  hunched | resp. ↑; less active; loss of appetite; piloerection; pale appearance | resp. ↑; less active; loss of appetite |
| **CM9** | resp. ↑ | resp. ↑; less active; loss of appetite;  hunched; needs more prompting to move | resp. ↑↑; less active; loss of appetite; hunched; needs more prompting to move; very shallow breathing | resp. ↑↑; anorexic; hunched; dull expression; no response to human presence; impossible to prompt to move; labored breathing; pale/blue mucus membranes | N/A | N/A |

^1^resp. ↑: >100 respirations/minute. ^2^N/A: not applicable (animal euthanized before this time point). ^3^resp. ↑↑: >150 respirations/minute. ^4^-: no change from baseline.
