# Supplementary material for: Infection with MERS-CoV Causes Lethal Pneumonia in the Common Marmoset
Source: PLoS Pathog. 2014 Aug 21;10(8):e1004250. doi: 10.1371/journal.ppat.1004250 (PMC4140844; doi:10.1371/journal.ppat.1004250)
Supplement: Table S2 — Clinical score sheet for common marmosets inoculated with MERS-CoV. (DOCX) [file ppat.1004250.s004.docx]

**Table S2.** Clinical score sheet for common marmosets inoculated with MERS-CoV.

|  | **Score** |  |
| --- | --- | --- |
| **General Appearance** |  |  |
| Normal and alert, moving without prompting | 0 |  |
| Slow/ quiet, hunched, but alert, interested, moving without prompting | 5 |  |
| Quieter, hunched ,but alert ,moving needs a lot of prompting | 10 |  |
| Loss of interest, almost impossible to prompt to move, dull expression  falling asleep while watched, little or no response to human presence | 15 |  |
|  |  |  |
| **Skin and Fur** |  |  |
| Normal | 0 |  |
| Pilo erection/ unkept appearance | 5 |  |
|  |  |  |
| **Discharges** |  |  |
| Oral/ nasal/ ocular | 5 |  |
|  |  |  |
| **Respiration** |  |  |
| Normal (60-100 bpm^1^) | 0 |  |
| Increased (100 -120 bpm) | 5 |  |
| Severely increased (> 120 bpm) , labored, cough, open mouth breathing | 10 |  |
| Dyspnea, cyanosis, foam | 15 |  |
|  |  |  |
| **Food consumption** |  |  |
| Loss of appetite | 2 |  |
| Anorexia | 5 |  |
|  | **Total^2^** |  |

^1^Breaths per minute. ^2^Euthanasia is indicated at a clinical score of 35.
