# Supplementary material for: Infection with MERS-CoV Causes Lethal Pneumonia in the Common Marmoset
Source: PLoS Pathog. 2014 Aug 21;10(8):e1004250. doi: 10.1371/journal.ppat.1004250 (PMC4140844; doi:10.1371/journal.ppat.1004250)
Supplement: Table S3 — Summary of radiographic changes of common marmosets inoculated with MERS-CoV. The quality and location of interstitial infiltration observed from ventral-dorsal and lateral x-rays is indicated. (DOCX) [file ppat.1004250.s005.docx]

**Table S3.** Summary of radiographic changes of common marmosets inoculated with MERS-CoV. The quality and location of interstitial infiltration observed from ventral-dorsal and lateral x-rays is indicated.

|  | **Day 1** | **Day 3** | **Day 4** | **Day 6** | **Day 9** | **Day 13** | **Day 20** |
| --- | --- | --- | --- | --- | --- | --- | --- |
| **CM1** | ND^1^ | RL^2^: mild diffuse LL^3^: mild diffuse | N/A^4^ | N/A | N/A | N/A | N/A |
| **CM2** | ND | RL: middle  LL: middle | N/A | N/A | N/A | N/A | N/A |
| **CM3** | ND | RL: middle diffuse  LL: middle diffuse | N/A | N/A | N/A | N/A | N/A |
| **CM4** | RL: marked diffuse  LL: marked diffuse | RL: middle linear diffuse  LL: middle linear diffuse | N/A | RL: severe diffuse  LL: severe diffuse | N/A | N/A | N/A |
| **CM5** | RL: mild  LL: mild | RL: middle  LL: middle | All lobes: severe, congested bronchioles | N/A | N/A | N/A | N/A |
| **CM6** | RL: mild  LL: mild | RL: severe  LL: severe | N/A | All lobes: severe, congested bronchioles | N/A | N/A | N/A |
| **CM7** | ND | RL: mild  LL: mild | N/A | RL: mild  LL: mild | RL: mild  LL: mild | RL: mild  LL: mild | ND |
| **CM8** | ND | RL: mild | N/A | RL: mild | RL: mild | RL: mild | ND |
| **CM9** | RL: mild  LL: mild | RL: severe  LL: severe | All lobes: severe, congested (complete) bronchioles | N/A | N/A | N/A | N/A |

^1^ND: no difference detected between baseline and this time point. ^2^RL: right lung lower lobe. ^3^LL: left lung lower lobe. ^4^N/A: animal was euthanized before this time point
