# Supplementary material for: Infection with MERS-CoV Causes Lethal Pneumonia in the Common Marmoset
Source: PLoS Pathog. 2014 Aug 21;10(8):e1004250. doi: 10.1371/journal.ppat.1004250 (PMC4140844; doi:10.1371/journal.ppat.1004250)
Supplement: Table S4 — Histopathology score in MERS-CoV inoculated common marmosets based on area of tissues affected by microscopic lesions. (DOCX) [file ppat.1004250.s006.docx]

**Table S4.** Histopathology score in MERS-CoV inoculated common marmosets based on area of tissues affected by microscopic lesions.

|  | **3 dpi** | | | **4 dpi** | | **6 dpi** | |
| --- | --- | --- | --- | --- | --- | --- | --- |
|  | **CM1** | **CM2** | **CM3** | **CM5** | **CM9** | **CM4** | **CM6** |
| **Conjunctiva** | 0 | 0 | 0 | 0 | 0 | 0 | 0 |
| **Nasal mucosa** | 0 | 0 | 0 | 0 | 0 | 0 | 0 |
| **Oropharynx** | 0 | 0 | 0 | 0 | 0 | 0 | 0 |
| **Trachea** | 0 | 0 | 0 | 0 | 0 | 0 | 0 |
| **Right lung, upper lobe** |  |  |  |  |  |  |  |
| bronchointerstitial pneumonia, acute, with fibrin and edema | 0 | 3 | 3 | 3 | 3 | 2 | 4 |
| type II pneumocyte hyperplasia | 0 | 0 | 0 | 0 | 3 | 3 | 3 |
| **Right lung, lower lobe** |  |  |  |  |  |  |  |
| bronchointerstitial pneumonia, acute, with fibrin and edema | 4 | 4 | 3 | 3 | 4 | 4 | 4 |
| type II pneumocyte hyperplasia | 0 | 0 | 0 | 0 | 2 | 3 | 3 |
| **Left lung, upper lobe** |  |  |  |  |  |  |  |
| bronchointerstitial pneumonia, acute, with fibrin and edema | 2 | 3 | 2 | 3 | 3 | 3 | 0 |
| type II pneumocyte hyperplasia | 0 | 0 | 0 | 0 | 2 | 3 | 0 |
| **Left lung, lower lobe** |  |  |  |  |  |  |  |
| bronchointerstitial pneumonia, acute, with fibrin and edema | 3 | 4 | 4 | 3 | 4 | 4 | 4 |
| type II pneumocyte hyperplasia | 0 | 0 | 0 | 0 | 1 | 3 | 3 |

Score: 0: no pathologic changes; 1: few inflammatory foci scattered between multiple lung lobes; alveolar interstitium is minimally thickened by congestion and small numbers of neutrophils and macrophages; few neutrophils and macrophages within alveoli; 2: multiple inflammatory foci present in multiple lung lobes; alveolar interstitium is mildly thickened by congestion, edema and small numbers of neutrophils and macrophages; small numbers of neutrophils and macrophages within alveoli; 3: multiple inflammatory foci scattered within single lung lobe; alveolar interstitium is moderately thickened by congestion, edema and moderate numbers of neutrophils and macrophages; many neutrophils and macrophages within alveoli; small amounts of fibrin and edema in alveoli; 4: multiple to coalescing inflammatory foci within a single lung lobe; alveolar interstitium is markedly thickened by congestion, edema, fibrin and large numbers of neutrophils and macrophages; large numbers of neutrophils, macrophages, cellular debris, fibrin and edema within alveoli.
