# Supplementary material for: Infection with MERS-CoV Causes Lethal Pneumonia in the Common Marmoset
Source: PLoS Pathog. 2014 Aug 21;10(8):e1004250. doi: 10.1371/journal.ppat.1004250 (PMC4140844; doi:10.1371/journal.ppat.1004250)
Supplement: Table S5 — Virus isolation from tissues of common marmosets inoculated with MERS-CoV in VeroE6 or LLC-MK2 cells. (DOCX) [file ppat.1004250.s007.docx]

**Table S5.** Virus isolation from tissues of common marmosets inoculated with MERS-CoV in VeroE6 or LLC-MK2 cells.

| Tissue | Virus isolation | | | | | | |
| --- | --- | --- | --- | --- | --- | --- | --- |
|  | 3 dpi | | | 4 dpi | | 6 dpi | |
|  | CM1 | CM2 | CM3 | CM5 | CM9 | CM4 | CM6 |
| nasal mucosa | + | - | - | - | - | - | - |
| trachea | - | - | - | + | + | + | + |
| RU lobe | - | + | + | - | + | - | - |
| RL lobe | + | + | + | + | + | + | - |
| LU lobe | + | + | + | - | + | - | - |
| LL lobe | + | + | + | + | + | - | - |
| kidney | - | - | - | - | - | - | - |

RU: right upper lung lobe; RL: right lower lung lobe; LU: left upper lung lobe; RL: left lower lung lobe

+ virus isolated from tissue

- virus not isolated from tissue
