# Supplementary material for: Infection with MERS-CoV Causes Lethal Pneumonia in the Common Marmoset
Source: PLoS Pathog. 2014 Aug 21;10(8):e1004250. doi: 10.1371/journal.ppat.1004250 (PMC4140844; doi:10.1371/journal.ppat.1004250)
Supplement: Table S6 — Differentially expressed genes identified to be involved in fibrosis pathways. (DOCX) [file ppat.1004250.s008.docx]

**Table S6.** Differentially expressed genes identified to be involved in fibrosis pathways.

| **Symbol** | **Entrez Gene Name** | **Human Ortholog Ensembl Identifier** | **Log_2_ ratio to uninfected** | | | | | | | | | |
| --- | --- | --- | --- | --- | --- | --- | --- | --- | --- | --- | --- | --- |
|  |  |  | **CM1** | **CM2** | **CM3** | **3 dpi mean** | **CM5** | **CM9** | **4 dpi mean** | **CM4** | **CM6** | **6 dpi mean** |
| ADAM12 | ADAM metallopeptidase domain 12 | ENSG00000148848 | 0.7059 | 1.9850 | 2.3299 | 1.6736 | 2.1521 | 2.9934 | 2.2014 | 1.5577 | 1.1447 | 1.4587 |
| AGT | angiotensinogen (serpin peptidase inhibitor, clade A, member 8) | ENSG00000135744 | 1.1912 | 1.1056 | 1.2995 | 1.1987 | 1.6737 | 1.8397 | 1.5717 | 0.5891 | 1.8173 | 1.2017 |
| AKAP12 | A kinase (PRKA) anchor protein 12 | ENSG00000131016 | 1.0821 | 1.7063 | 1.1068 | 1.2984 | 1.3405 | 1.9649 | 1.5042 | 0.9933 | 1.3303 | 1.2073 |
| BMP2 | bone morphogenetic protein 2 | ENSG00000125845 | 1.2836 | 2.6433 | 2.8370 | 2.2546 | 2.4988 | 2.8046 | 2.4775 | 2.2748 | 1.8579 | 2.1291 |
| CALR | calreticulin | ENSG00000179218 | 0.7544 | 2.4706 | 2.1795 | 1.8015 | 1.9783 | 1.8450 | 1.8325 | 1.5860 | 1.6352 | 1.6742 |
| CCL2 | chemokine (C-C motif) ligand 2 | ENSG00000108691 | 3.5348 | 2.6846 | 3.7765 | 3.3320 | 1.0813 | 3.9117 | 2.8708 | 3.5928 | 3.9340 | 3.6196 |
| CD4 | CD4 molecule | ENSG00000010610 | 1.3708 | 3.3164 | 2.2619 | 2.3164 | 1.4972 | 1.6831 | 1.6724 | 1.4708 | 1.7234 | 1.8368 |
| CD46 | CD46 molecule, complement regulatory protein | ENSG00000117335 | -1.8527 | -1.3300 | -1.6849 | -1.6225 | -1.2987 | -0.3732 | -1.1007 | -1.4477 | -1.8206 | -1.6303 |
| CDH3 | cadherin 3, type 1, P-cadherin (placental) | ENSG00000062038 | 0.6040 | 2.5545 | 0.3022 | 1.1536 | 2.8498 | 3.1392 | 2.5469 | 2.5304 | 1.2712 | 1.6517 |
| COL11A2 | collagen, type XI, alpha 2 | ENSG00000204248 | 0.2112 | 1.1447 | 1.6122 | 0.9894 | 3.1719 | 1.8077 | 2.0121 | -0.4227 | 2.6029 | 1.0565 |
| COL13A1 | collagen, type XIII, alpha 1 | ENSG00000197467 | 1.6744 | 0.9415 | 2.0727 | 1.5628 | 1.4127 | 1.3100 | 1.1247 | -0.5592 | 0.9509 | 0.6515 |
| COL14A1 | collagen, type XIV, alpha 1 | ENSG00000187955 | 0.3234 | 1.5463 | 1.3687 | 1.0794 | 1.2339 | 1.4184 | 1.2059 | 0.3992 | 1.4173 | 0.9653 |
| COL15A1 | collagen, type XV, alpha 1 | ENSG00000204291 | 2.3641 | 3.4164 | 2.8078 | 2.8628 | 3.7984 | 4.1888 | 3.5950 | 2.4141 | 3.1166 | 2.7978 |
| COL16A1 | collagen, type XVI, alpha 1 | ENSG00000084636 | 1.4986 | 3.0756 | 3.0798 | 2.5514 | 3.2207 | 3.6311 | 2.9374 | 0.2350 | 3.0952 | 1.9605 |
| COL18A1 | collagen, type XVIII, alpha 1 | ENSG00000182871 | -0.1766 | 2.4719 | 2.1298 | 1.4751 | 2.7287 | 3.4552 | 2.4798 | -0.2170 | 2.5086 | 1.2555 |
| COL1A1 | collagen, type I, alpha 1 | ENSG00000108821 | 3.5644 | 7.0682 | 6.2317 | 5.6214 | 6.0792 | 7.2749 | 6.2258 | 3.6969 | 6.6515 | 5.3233 |
| COL1A2 | collagen, type I, alpha 2 | ENSG00000164692 | 3.9724 | 5.6617 | 5.4792 | 5.0378 | 5.0878 | 5.5317 | 5.1032 | 3.7816 | 5.2509 | 4.6901 |
| COL20A1 | collagen, type XX, alpha 1 | ENSG00000101203 | 0.4292 | 1.9008 | 1.5109 | 1.2803 | 1.8772 | 2.5858 | 1.7526 | -0.7275 | 1.8314 | 0.7948 |
| COL23A1 | collagen, type XXIII, alpha 1 | ENSG00000050767 | 0.6660 | 0.4781 | 1.8894 | 1.0112 | 0.9690 | 1.2461 | 0.7328 | -1.0061 | -0.0551 | -0.0167 |
| COL3A1 | collagen, type III, alpha 1 | ENSG00000168542 | 2.5710 | 4.6461 | 4.3483 | 3.8551 | 3.1636 | 4.7950 | 3.8405 | 2.8827 | 3.9512 | 3.5630 |
| COL4A1 | collagen, type IV, alpha 1 | ENSG00000187498 | 1.4455 | 3.9193 | 3.1221 | 2.8289 | 3.1270 | 4.2219 | 3.2645 | 1.5210 | 2.9842 | 2.4447 |
| COL4A2 | collagen, type IV, alpha 2 | ENSG00000134871 | 0.8026 | 3.1748 | 2.4052 | 2.1275 | 2.9235 | 4.1142 | 2.9401 | 0.3874 | 2.8327 | 1.7826 |
| COL4A4 | collagen, type IV, alpha 4 | ENSG00000081052 | 2.4051 | 2.5570 | 1.7209 | 2.2276 | 2.6097 | 2.6600 | 2.2269 | 1.0894 | 0.9165 | 1.4112 |
| COL5A1 | collagen, type V, alpha 1 | ENSG00000130635 | 0.9350 | 2.9241 | 2.7661 | 2.2084 | 2.7519 | 3.8320 | 2.7856 | 0.2292 | 2.8810 | 1.7729 |
| COL5A2 | collagen, type V, alpha 2 | ENSG00000204262 | 1.4397 | 2.4804 | 2.2594 | 2.0598 | 2.4770 | 3.7675 | 2.8692 | 1.9443 | 3.0847 | 2.3630 |
| COL5A3 | collagen, type V, alpha 3 | ENSG00000080573 | 1.4954 | 4.0230 | 3.5990 | 3.0391 | 3.1020 | 4.9339 | 3.5086 | 0.9036 | 3.5267 | 2.4898 |
| COL6A1 | collagen, type VI, alpha 1 | ENSG00000142156 | 1.3808 | 3.5695 | 3.4832 | 2.8112 | 3.2140 | 4.8317 | 3.4267 | 0.2703 | 3.6218 | 2.2344 |
| COL6A2 | collagen, type VI, alpha 2 | ENSG00000142173 | 0.9095 | 3.5261 | 3.4275 | 2.6210 | 3.2541 | 4.4483 | 3.2195 | 0.0372 | 3.2101 | 1.9561 |
| COL6A3 | collagen, type VI, alpha 3 | ENSG00000163359 | 2.4978 | 4.2467 | 3.9606 | 3.5684 | 3.4574 | 3.8857 | 3.5053 | 2.2511 | 3.6989 | 3.1728 |
| COL7A1 | collagen, type VII, alpha 1 | ENSG00000114270 | 0.5843 | 2.8348 | 3.0302 | 2.1497 | 3.1382 | 4.4890 | 3.2951 | 1.4955 | 3.1296 | 2.2583 |
| COL8A1 | collagen, type VIII, alpha 1 | ENSG00000144810 | 0.1963 | -0.1176 | 0.3511 | 0.1433 | 1.7290 | 2.0325 | 1.2520 | 0.1072 | -0.2665 | -0.0053 |
| COLGALT1 | collagen beta(1-O)galactosyltransferase 1 | ENSG00000130309 | 0.3746 | 2.4378 | 2.3443 | 1.7189 | 2.3358 | 2.6921 | 1.9993 | -0.3810 | 1.5721 | 0.9700 |
| CSF1 | colony stimulating factor 1 (macrophage) | ENSG00000184371 | 1.1404 | 3.2525 | 3.7785 | 2.7238 | 2.3896 | 4.6379 | 3.1293 | 0.8640 | 3.4936 | 2.3605 |
| CSK | c-src tyrosine kinase | ENSG00000103653 | -0.1431 | 1.8538 | 0.9262 | 0.8789 | 1.1187 | 1.8512 | 1.2129 | -0.4030 | 1.5306 | 0.6688 |
| CYR61 | cysteine-rich, angiogenic inducer, 61 | ENSG00000142871 | 2.3746 | 4.7359 | 3.7094 | 3.6066 | 4.6837 | 4.8750 | 4.2697 | 3.0688 | 3.0761 | 3.2505 |
| FIGF | c-fos induced growth factor (vascular endothelial growth factor D) | ENSG00000165197 | -0.3211 | -2.1523 | -1.8042 | -1.4259 | -1.1015 | -2.2515 | -1.6406 | -1.7369 | -1.5433 | -1.5687 |
| FN1 | fibronectin 1 | ENSG00000115414 | 1.8433 | 1.8893 | 1.4338 | 1.7221 | 1.8731 | 2.1626 | 2.0140 | 2.0998 | 2.1968 | 2.0062 |
| HRAS | Harvey rat sarcoma viral oncogene homolog | ENSG00000174775 | 0.4555 | 1.3080 | 0.9362 | 0.8999 | 2.3141 | 2.2970 | 1.8561 | 0.4806 | 1.4910 | 0.9572 |
| ITGA5 | integrin, alpha 5 (fibronectin receptor, alpha polypeptide) | ENSG00000161638 | 0.2515 | 1.0513 | 0.7859 | 0.6963 | 1.0974 | 1.5741 | 1.1004 | -0.0462 | 1.2387 | 0.6296 |
| ITGAM | integrin, alpha M (complement component 3 receptor 3 subunit) | ENSG00000125730 | 0.5815 | 0.4513 | 0.6656 | 0.5661 | 0.5977 | 0.7490 | 0.6611 | 0.6004 | 0.7435 | 0.6367 |
| ITGAV | integrin, alpha V | ENSG00000138448 | -0.7312 | -2.1583 | -1.9365 | -1.6087 | -1.5212 | -1.6255 | -1.4500 | -0.2055 | -1.7955 | -1.2032 |
| ITGB1 | integrin, beta 1 (fibronectin receptor, beta polypeptide, antigen CD29 includes MDF2, MSK12) | ENSG00000150093 | -0.4901 | -1.8764 | -1.9824 | -1.4497 | -1.8071 | -2.0045 | -1.6515 | -0.2535 | -1.7255 | -1.1429 |
| JUP | junction plakoglobin | ENSG00000173801 | 0.7358 | 1.5954 | 1.4732 | 1.2681 | 2.3704 | 2.1968 | 1.8709 | 0.4384 | 1.4304 | 1.0456 |
| LAMC3 | laminin, gamma 3 | ENSG00000050555 | 1.1145 | 1.5496 | 1.6886 | 1.4509 | 2.1487 | 3.8395 | 2.4643 | -0.3350 | 3.0984 | 1.4048 |
| LDLR | low density lipoprotein receptor | ENSG00000130164 | 1.8722 | 2.9820 | 2.8970 | 2.5837 | 3.0073 | 4.3169 | 3.1547 | 1.2290 | 2.6063 | 2.1397 |
| LGALS8 | lectin, galactoside-binding, soluble, 8 | ENSG00000116977 | -0.5390 | -0.6659 | -0.9761 | -0.7270 | -1.1886 | -1.6526 | -1.2252 | -0.5719 | -1.2042 | -0.8344 |
| LRP1 | low density lipoprotein receptor-related protein 1 | ENSG00000123384 | 0.5058 | 1.1111 | 0.6341 | 0.7503 | 1.4455 | 1.4702 | 1.0954 | -1.0971 | 1.4584 | 0.3705 |
| LTBP2 | latent transforming growth factor beta binding protein 2 | ENSG00000119681 | 0.0789 | 0.7979 | 0.0781 | 0.3183 | 1.1336 | 1.8022 | 1.0997 | -0.4170 | 1.1887 | 0.3633 |
| MAP3K1 | mitogen-activated protein kinase kinase kinase 1, E3 ubiquitin protein ligase | ENSG00000095015 | -0.6906 | -1.4170 | -1.6382 | -1.2486 | -1.8309 | -2.2307 | -1.7728 | -0.8750 | -1.6470 | -1.2569 |
| MAPK3 | mitogen-activated protein kinase 3 | ENSG00000102882 | 0.6702 | 1.8553 | 1.4065 | 1.3107 | 2.0385 | 2.4128 | 1.6593 | -0.7668 | 1.0355 | 0.5265 |
| MMP2 | matrix metallopeptidase 2 (gelatinase A, 72kDa gelatinase, 72kDa type IV collagenase) | ENSG00000087245 | 2.3425 | 3.5392 | 2.9455 | 2.9424 | 3.7171 | 3.4199 | 3.3871 | 2.4363 | 3.6942 | 3.0243 |
| MYOC | myocilin, trabecular meshwork inducible glucocorticoid response | ENSG00000034971 | -1.3781 | -2.1008 | -0.6968 | -1.3919 | -1.1127 | -1.3572 | -1.2621 | -1.7674 | -0.7901 | -1.3165 |
| PDCD6IP | programmed cell death 6 interacting protein | ENSG00000170248 | -0.4529 | -1.8530 | -1.5551 | -1.2870 | -1.8525 | -1.8594 | -1.5516 | -0.3518 | -1.1900 | -0.9429 |
| PLAUR | plasminogen activator, urokinase receptor | ENSG00000011422 | 0.1861 | 1.1130 | 1.2423 | 0.8471 | 1.1563 | 2.3319 | 1.4452 | 0.9707 | 0.7246 | 0.8475 |
| PLEC | plectin | ENSG00000178209 | 0.0957 | 2.0312 | 1.8084 | 1.3118 | 2.4102 | 3.1436 | 2.1641 | -0.7323 | 2.2359 | 0.9385 |
| PML | promyelocytic leukemia | ENSG00000140464 | 2.4884 | 3.5940 | 3.2302 | 3.1042 | 2.8093 | 3.5167 | 2.9737 | 1.1511 | 3.5300 | 2.5951 |
| PTK2B | protein tyrosine kinase 2 beta | ENSG00000120899 | 0.4369 | 1.5167 | 1.5831 | 1.1789 | 1.3978 | 1.3588 | 1.3188 | 0.7844 | 1.6363 | 1.1999 |
| RAB21 | RAB21, member RAS oncogene family | ENSG00000080371 | -0.7641 | -1.5896 | -1.2433 | -1.1990 | -1.5938 | -1.1515 | -1.2444 | -0.3463 | -1.4185 | -0.9879 |
| RELA | v-rel avian reticuloendotheliosis viral oncogene homolog A | ENSG00000173039 | 0.3253 | 1.8776 | 1.6808 | 1.2946 | 1.6014 | 2.3019 | 1.5957 | -0.2718 | 1.6287 | 0.8838 |
| RHOD | ras homolog family member D | ENSG00000173156 | 0.4830 | 1.9676 | 1.9984 | 1.4830 | 2.5413 | 3.1632 | 2.2540 | 0.3587 | 1.3308 | 1.0575 |
| ROCK1 | Rho-associated, coiled-coil containing protein kinase 1 | ENSG00000067900 | -0.5116 | -0.9606 | -1.3993 | -0.9572 | -1.5101 | -1.0836 | -1.1587 | -0.1011 | -1.5893 | -0.8825 |
| ROCK2 | Rho-associated, coiled-coil containing protein kinase 2 | ENSG00000134318 | -0.6045 | -2.2372 | -1.5952 | -1.4789 | -1.7323 | -1.3232 | -1.3953 | -0.0881 | -1.8247 | -1.1306 |
| RPL22 | ribosomal protein L22 | ENSG00000116251 | -0.9583 | -0.9219 | -1.0695 | -0.9832 | -1.0836 | -2.0055 | -1.5446 | -0.5549 | -0.9119 | -0.7334 |
| RUNX2 | runt-related transcription factor 2 | ENSG00000124813 | -0.8340 | -0.9345 | -1.1491 | -0.9725 | -2.1466 | -0.6086 | -1.1413 | 0.0365 | -1.0699 | -0.6687 |
| SPP1 | secreted phosphoprotein 1 | ENSG00000118785 | 0.2368 | -1.9266 | -1.2814 | -0.9904 | -2.0512 | -0.8804 | -1.1556 | 0.9267 | -1.5416 | -0.5351 |
| SRC | v-src avian sarcoma (Schmidt-Ruppin A-2) viral oncogene homolog | ENSG00000197122 | 1.3335 | 2.8395 | 2.4376 | 2.2035 | 3.3040 | 3.6877 | 3.0239 | 0.9435 | 3.0931 | 2.0800 |
| TGFB1 | transforming growth factor, beta 1 | ENSG00000105329 | 0.1228 | 2.2266 | 2.1777 | 1.5091 | 1.4660 | 2.2567 | 1.5363 | -0.7165 | 1.8665 | 0.8863 |
| TGFBI | transforming growth factor, beta-induced, 68kDa | ENSG00000120708 | 0.6328 | 1.2714 | 0.6786 | 0.8609 | 0.7723 | 1.2255 | 1.1619 | 1.7873 | 1.8159 | 1.4880 |
| TIMP2 | TIMP metallopeptidase inhibitor 2 | ENSG00000035862 | 1.2621 | 1.3790 | 1.2772 | 1.3061 | 1.6090 | 1.3758 | 1.3702 | 0.5796 | 1.4916 | 1.1258 |
| TLN2 | talin 2 | ENSG00000171914 | 0.7057 | 1.1437 | 0.5828 | 0.8107 | 2.0374 | 2.0415 | 1.7107 | 1.0606 | 1.2885 | 1.0533 |
| TNC | tenascin C | ENSG00000041982 | 2.4119 | 4.3553 | 2.7411 | 3.1695 | 3.0371 | 5.0523 | 4.0550 | 3.7673 | 5.2905 | 4.0757 |
| TNFRSF11B | tumor necrosis factor receptor superfamily, member 11b | ENSG00000164761 | -1.0038 | -1.5516 | -1.7214 | -1.4256 | -2.6321 | -2.0640 | -2.1802 | -1.7834 | -2.3241 | -1.8444 |
| VEGFC | vascular endothelial growth factor C | ENSG00000150630 | -0.9674 | -2.3388 | -1.4365 | -1.5809 | -1.9693 | -1.6518 | -1.6404 | -0.9677 | -1.3514 | -1.3000 |
| ZYX | zyxin | ENSG00000159840 | 1.0282 | 2.6800 | 2.5456 | 2.0846 | 2.2022 | 3.8996 | 2.5470 | -0.0211 | 2.5543 | 1.5393 |
