# Supplementary material for: Infection with MERS-CoV Causes Lethal Pneumonia in the Common Marmoset
Source: PLoS Pathog. 2014 Aug 21;10(8):e1004250. doi: 10.1371/journal.ppat.1004250 (PMC4140844; doi:10.1371/journal.ppat.1004250)
Supplement: Table S7 — Differentially expressed genes identified to be involved in antiviral pathways. (DOCX) [file ppat.1004250.s009.docx]

**Table S7.** Differentially expressed genes identified to be involved in antiviral pathways.

| **Symbol** | **Entrez Gene Name** | **Human Ortholog Ensembl Identifier** | **Log_2_ ratio to uninfected** | | | | | | | | | |
| --- | --- | --- | --- | --- | --- | --- | --- | --- | --- | --- | --- | --- |
|  |  |  | **CM1** | **CM2** | **CM3** | **3 dpi mean** | **CM5** | **CM9** | **4 dpi mean** | **CM4** | **CM6** | **6 dpi mean** |
| CCL18 | chemokine (C-C motif) ligand 18 (pulmonary and activation-regulated) |  |  |  |  |  |  |  |  |  |  |  |
| CCL2 | chemokine (C-C motif) ligand 2 | ENSG00000108691 | 3.5348 | 2.6846 | 3.7765 | 3.3320 | 1.0813 | 3.9117 | 2.8708 | 3.5928 | 3.9340 | 3.6196 |
| CCL21 | chemokine (C-C motif) ligand 21 | ENSG00000137077 | 1.7734 | 1.4308 | 2.0356 | 1.7466 | 2.2602 | 1.0525 | 1.4355 | -0.4601 | 1.6950 | 0.9939 |
| CCL3 | chemokine (C-C motif) ligand 3 |  |  |  |  |  |  |  |  |  |  |  |
| CCL5 | chemokine (C-C motif) ligand 5 | ENSG00000161570 | -3.7287 | -2.0251 | -2.9081 | -2.8873 | -2.7808 | -4.5908 | -3.4970 | -3.9813 | -2.4894 | -3.1193 |
| CD40LG | CD40 ligand |  |  |  |  |  |  |  |  |  |  |  |
| CSF1 | colony stimulating factor 1 (macrophage) | ENSG00000184371 | 1.1404 | 3.2525 | 3.7785 | 2.7238 | 2.3896 | 4.6379 | 3.1293 | 0.8640 | 3.4936 | 2.3605 |
| CSF2 | colony stimulating factor 2 (granulocyte-macrophage) |  |  |  |  |  |  |  |  |  |  |  |
| CSF3 | colony stimulating factor 3 (granulocyte) |  |  |  |  |  |  |  |  |  |  |  |
| CX3CL1 | chemokine (C-X3-C motif) ligand 1 | ENSG00000006210 | 0.9445 | 3.0109 | 2.8911 | 2.2822 | 2.2660 | 2.5641 | 2.2061 | 0.4021 | 2.6805 | 1.7883 |
| CXCL12 | chemokine (C-X-C motif) ligand 12 | ENSG00000107562 | 0.7646 | 1.8834 | 1.9614 | 1.5365 | 1.8250 | 1.2631 | 1.5642 | 1.4084 | 1.8688 | 1.6046 |
| CXCL16 | chemokine (C-X-C motif) ligand 16 | ENSG00000161921 | 1.1188 | 1.3468 | 0.8899 | 1.1185 | 1.1851 | 1.3615 | 1.3264 | 1.1779 | 2.0018 | 1.4327 |
| DDX58 | DEAD (Asp-Glu-Ala-Asp) box polypeptide 58 | ENSG00000107201 | 3.4999 | 1.9231 | 2.2788 | 2.5673 | 0.7397 | 0.8464 | 1.3319 | 2.9590 | 1.7027 | 2.4096 |
| FLT3LG | fms-related tyrosine kinase 3 ligand | ENSG00000090554 | 0.4555 | 2.1946 | 2.3198 | 1.6566 | 2.2871 | 2.5036 | 2.3954 | -0.4851 | 2.1963 | 0.8556 |
| Gm-csf |  |  |  |  |  |  |  |  |  |  |  |  |
| Gm-Csf Receptor |  |  |  |  |  |  |  |  |  |  |  |  |
| IFN alpha/beta |  |  |  |  |  |  |  |  |  |  |  |  |
| IFN Beta |  |  |  |  |  |  |  |  |  |  |  |  |
| Ifn gamma |  |  |  |  |  |  |  |  |  |  |  |  |
| IFN type 1 |  |  |  |  |  |  |  |  |  |  |  |  |
| IFN1a | interferon, type 1, cluster |  |  |  |  |  |  |  |  |  |  |  |
| Ifna | interferon alpha |  |  |  |  |  |  |  |  |  |  |  |
| IFNA1/IFNA13 | interferon, alpha 1 |  |  |  |  |  |  |  |  |  |  |  |
| IFNA2 | interferon, alpha 2 |  |  |  |  |  |  |  |  |  |  |  |
| IFNAR1 | interferon (alpha, beta and omega) receptor 1 | ENSG00000142166 | 0.1190 | 1.4073 | 1.4497 | 0.9920 | 1.2645 | 1.4632 | 1.3639 | 0.3385 | 0.5506 | 0.4445 |
| IFNAR2 | interferon (alpha, beta and omega) receptor 2 | ENSG00000249624 | 0.0200 | 0.2258 | -0.0354 | 0.0701 | 0.1193 | -0.2185 | -0.0496 | 0.4364 | 0.5758 | 0.5061 |
| IFNB1 | interferon, beta 1, fibroblast |  |  |  |  |  |  |  |  |  |  |  |
| IFNE | interferon, epsilon |  |  |  |  |  |  |  |  |  |  |  |
| IFNG | interferon, gamma | ENSG00000111537 | -2.3715 | -1.6491 | -1.6814 | -1.9007 | -2.5479 | -2.5479 | -2.2172 | -1.3461 | -1.4206 | -1.5558 |
| IFNGR1 | interferon gamma receptor 1 | ENSG00000027697 | -1.5385 | -4.7224 | -3.8158 | -3.3589 | -3.2845 | -4.1583 | -3.3739 | -1.4723 | -3.2053 | -2.6788 |
| IFNGR2 | interferon gamma receptor 2 (interferon gamma transducer 1) | ENSG00000159128 | 0.0378 | 0.6588 | 0.5940 | 0.4302 | 0.1536 | 0.3823 | 0.4083 | 0.6366 | 1.0006 | 0.6891 |
| IFNLR1 | interferon, lambda receptor 1 | ENSG00000185436 | 1.4101 | 2.5720 | 2.4338 | 2.1386 | 2.5340 | 2.8474 | 2.3818 | 1.5534 | 1.6005 | 1.7642 |
| IK | IK cytokine, down-regulator of HLA II | ENSG00000113141 | 0.4730 | 1.7353 | 1.8127 | 1.3404 | 1.3344 | 1.1822 | 1.0924 | 0.3460 | 0.5952 | 0.7605 |
| IL1 |  |  |  |  |  |  |  |  |  |  |  |  |
| IL12 (complex) |  |  |  |  |  |  |  |  |  |  |  |  |
| IL12RB2 | interleukin 12 receptor, beta 2 | ENSG00000081985 | -1.3419 | -1.2862 | -0.6327 | -1.0869 | -1.2903 | -1.5027 | -1.1563 | -0.2961 | -0.6446 | -0.6759 |
| IL13 | interleukin 13 |  |  |  |  |  |  |  |  |  |  |  |
| IL17RC | interleukin 17 receptor C | ENSG00000163702 | 0.3757 | 1.7754 | 1.8020 | 1.3177 | 1.5653 | 2.2221 | 1.5120 | -0.4406 | 1.3686 | 0.7485 |
| IL17RE | interleukin 17 receptor E | ENSG00000163701 | 1.0154 | -0.4196 | -0.0058 | 0.1967 | 1.2597 | 1.2807 | 1.0157 | 0.7789 | 0.5449 | 0.5068 |
| IL18 | interleukin 18 (interferon-gamma-inducing factor) | ENSG00000150782 | -0.6448 | -1.8468 | -0.0520 | -0.8479 | -1.2697 | -1.8468 | -1.3215 | -0.2237 | -1.4726 | -0.8480 |
| IL18BP | interleukin 18 binding protein | ENSG00000137496 | 1.3060 | 3.4218 | 2.6319 | 2.4532 | 2.8356 | 2.7186 | 2.5425 | 0.6730 | 3.0939 | 2.0734 |
| IL18R1 | interleukin 18 receptor 1 | ENSG00000115604 | -1.5029 | -2.4470 | -1.5613 | -1.8371 | -2.1681 | -1.9570 | -1.9470 | -1.0165 | -2.2942 | -1.7159 |
| IL1A | interleukin 1, alpha |  |  |  |  |  |  |  |  |  |  |  |
| IL1B | interleukin 1, beta | ENSG00000125538 | -0.5249 | 0.1355 | -1.1546 | -0.5147 | -0.3370 | 0.3142 | 0.0187 | 0.1101 | 0.6413 | 0.0789 |
| IL1R1 | interleukin 1 receptor, type I | ENSG00000115594 | 0.1019 | -1.3976 | -1.3376 | -0.8778 | -1.8805 | -2.2389 | -1.4671 | 0.6641 | -0.6319 | -0.2818 |
| IL1RAP | interleukin 1 receptor accessory protein | ENSG00000196083 | -0.4644 | -1.5135 | -0.7191 | -0.8990 | -1.7743 | -1.4972 | -1.2856 | -0.0526 | -0.8048 | -0.5855 |
| IL1RN | interleukin 1 receptor antagonist |  |  |  |  |  |  |  |  |  |  |  |
| IL2 | interleukin 2 |  |  |  |  |  |  |  |  |  |  |  |
| IL20RA | interleukin 20 receptor, alpha | ENSG00000016402 | -1.0904 | -2.5452 | -1.6275 | -1.7543 | -1.9339 | -1.7201 | -1.7075 | -1.1232 | -1.5281 | -1.4685 |
| IL22RA1 | interleukin 22 receptor, alpha 1 | ENSG00000142677 | 0.9631 | 1.1041 | 1.2722 | 1.1131 | 1.4643 | 1.5845 | 1.2825 | 0.6388 | 0.6444 | 0.7988 |
| IL23A | interleukin 23, alpha subunit p19 |  |  |  |  |  |  |  |  |  |  |  |
| IL27 | interleukin 27 | ENSG00000197272 | 0.4788 | 3.9923 | 3.3471 | 2.6061 | 1.8518 | 2.7739 | 2.1121 | 0.3045 | 2.2210 | 1.7105 |
| IL27RA | interleukin 27 receptor, alpha | ENSG00000104998 | -0.3295 | 1.6705 | 1.8650 | 1.0687 | 0.7961 | 1.5181 | 1.0399 | 0.1730 | 1.1746 | 0.8054 |
| IL2RB | interleukin 2 receptor, beta | ENSG00000100385 | 0.6328 | 2.7713 | 2.0561 | 1.8201 | 2.5212 | 2.7713 | 2.3451 | 0.7860 | 2.6219 | 1.7427 |
| IL3 | interleukin 3 (colony-stimulating factor, multiple) |  |  |  |  |  |  |  |  |  |  |  |
| IL4 | interleukin 4 |  |  |  |  |  |  |  |  |  |  |  |
| IL4R | interleukin 4 receptor | ENSG00000077238 | 0.1005 | 1.4933 | 1.1714 | 0.9217 | 2.2522 | 2.8461 | 2.0958 | 0.3437 | 2.3023 | 1.1892 |
| IL5 | interleukin 5 (colony-stimulating factor, eosinophil) |  |  |  |  |  |  |  |  |  |  |  |
| IL6 | interleukin 6 (interferon, beta 2) |  |  |  |  |  |  |  |  |  |  |  |
| IL6R | interleukin 6 receptor | ENSG00000160712 | -0.4849 | 1.2345 | 1.6993 | 0.8163 | 1.4285 | 1.2699 | 1.0412 | -1.0935 | 1.5529 | 0.4252 |
| IL6ST | interleukin 6 signal transducer (gp130, oncostatin M receptor) | ENSG00000134352 | -0.6305 | -2.3675 | -2.3519 | -1.7833 | -1.9589 | -2.1271 | -1.7215 | -0.1578 | -1.2945 | -1.0785 |
| IL8 | interleukin 8 | ENSG00000169429 | -1.9556 | -2.3171 | -1.7220 | -1.9982 | -2.3600 | -1.9207 | -1.9168 | -0.7680 | -1.6428 | -1.4697 |
| Il8r |  |  |  |  |  |  |  |  |  |  |  |  |
| MIF | macrophage migration inhibitory factor (glycosylation-inhibiting factor) | ENSG00000240972 |  |  |  |  |  |  |  |  |  |  |
| Stat1 dimer |  |  |  |  |  |  |  |  |  |  |  |  |
| Stat3-Stat3 |  |  |  |  |  |  |  |  |  |  |  |  |
| Tap |  |  |  |  |  |  |  |  |  |  |  |  |
| Tgf beta |  |  |  |  |  |  |  |  |  |  |  |  |
| TGFA | transforming growth factor, alpha |  |  |  |  |  |  |  |  |  |  |  |
| TGFB1 | transforming growth factor, beta 1 | ENSG00000105329 | 0.1228 | 2.2266 | 2.1777 | 1.5091 | 1.4660 | 2.2567 | 1.5363 | -0.7165 | 1.8665 | 0.8863 |
| TGFB2 | transforming growth factor, beta 2 | ENSG00000092969 | 0.6617 | -0.9127 | -0.3518 | -0.2009 | 0.5481 | -0.1709 | 0.1943 | 1.2190 | -0.4014 | 0.2056 |
| TGFB3 | transforming growth factor, beta 3 | ENSG00000119699 | 0.6280 | 1.0025 | 0.6688 | 0.7664 | 0.9290 | 1.2994 | 1.0560 | 1.7063 | 0.3461 | 0.9396 |
| TGFBR1 | transforming growth factor, beta receptor 1 | ENSG00000106799 | -1.0371 | -2.2161 | -1.9834 | -1.7455 | -1.9539 | -2.2247 | -1.8466 | -0.4486 | -1.8891 | -1.3611 |
| TGFBR2 | transforming growth factor, beta receptor II (70/80kDa) | ENSG00000163513 | 0.4317 | 0.9185 | 0.4826 | 0.6109 | 0.5645 | 1.3802 | 0.7268 | -0.2683 | 0.3647 | 0.2358 |
| TGFBR3 | transforming growth factor, beta receptor III | ENSG00000069702 | -0.7826 | -1.1286 | -1.0773 | -0.9961 | -0.4254 | -1.2040 | -0.9426 | -1.3790 | -1.2202 | -1.1984 |
| TNF | tumor necrosis factor |  |  |  |  |  |  |  |  |  |  |  |
| Tnf (family) |  |  |  |  |  |  |  |  |  |  |  |  |
| TNFAIP2 | tumor necrosis factor, alpha-induced protein 2 | ENSG00000185215 | 0.5098 | 2.2126 | 2.2525 | 1.6583 | 2.1854 | 2.1009 | 1.8799 | -0.0543 | 2.4564 | 1.3535 |
| TNFAIP3 | tumor necrosis factor, alpha-induced protein 3 | ENSG00000118503 | 0.4642 | 1.4880 | 1.8324 | 1.2615 | 1.4524 | 1.5411 | 1.3741 | 0.7921 | 1.3328 | 1.1288 |
| TNFRSF10A | tumor necrosis factor receptor superfamily, member 10a | ENSG00000104689 | 0.5393 | 1.8524 | 1.8510 | 1.4143 | 1.6401 | 1.8128 | 1.5477 | 0.7597 | 1.3965 | 1.1901 |
| TNFRSF11B | tumor necrosis factor receptor superfamily, member 11b | ENSG00000164761 | -1.0038 | -1.5516 | -1.7214 | -1.4256 | -2.6321 | -2.0640 | -2.1802 | -1.7834 | -2.3241 | -1.8444 |
| TNFRSF12A | tumor necrosis factor receptor superfamily, member 12A | ENSG00000006327 | 0.7971 | 2.6490 | 2.7391 | 2.0617 | 2.2073 | 4.3261 | 2.7410 | 0.9595 | 2.0474 | 1.6896 |
| TNFRSF14 | tumor necrosis factor receptor superfamily, member 14 | ENSG00000157873 | -0.2393 | 1.0001 | 0.2935 | 0.3514 | 1.0050 | 1.7613 | 1.0160 | -0.5656 | 1.0595 | 0.2818 |
| TNFRSF1A | tumor necrosis factor receptor superfamily, member 1A | ENSG00000067182 | 0.6712 | 2.0236 | 1.9358 | 1.5436 | 1.4705 | 2.0545 | 1.5754 | 0.2107 | 1.8493 | 1.2012 |
| TNFRSF1B | tumor necrosis factor receptor superfamily, member 1B | ENSG00000028137 | 0.4752 | 3.4479 | 3.3530 | 2.4254 | 2.6717 | 3.3525 | 2.5160 | 0.0528 | 2.0929 | 1.5237 |
| TNFRSF8 | tumor necrosis factor receptor superfamily, member 8 | ENSG00000120949 | 0.5068 | -1.5761 | -1.3646 | -0.8113 | -2.7496 | -2.4384 | -1.9622 | -0.4343 | -0.8498 | -0.6985 |
| TNFSF10 | tumor necrosis factor (ligand) superfamily, member 10 | ENSG00000121858 | 1.3314 | 2.3226 | 2.1616 | 1.9385 | 2.1704 | 2.6440 | 2.2469 | 1.2937 | 2.5471 | 1.9264 |
| TNFSF11 | tumor necrosis factor (ligand) superfamily, member 11 |  | 0.2023 | 1.1982 | 0.6978 | 0.6994 | 1.1316 | 1.6878 | 1.0579 | -0.3099 | 0.6733 | 0.3543 |
| TNFSF12 | tumor necrosis factor (ligand) superfamily, member 12 | ENSG00000239697 | 0.7657 | 2.0109 | 1.6011 | 1.4592 | 1.7860 | 1.6923 | 1.4863 | 0.3606 | 1.1219 | 0.9806 |
| TNIP1 | TNFAIP3 interacting protein 1 | ENSG00000145901 | -0.9674 | -2.3388 | -1.4365 | -1.5809 | -1.9693 | -1.6518 | -1.6404 | -0.9677 | -1.3514 | -1.3000 |
| TYK2 | tyrosine kinase 2 | ENSG00000105397 |  |  |  |  |  |  |  |  |  |  |
| VEGFA | vascular endothelial growth factor A | ENSG00000112715 |  |  |  |  |  |  |  |  |  |  |
| VEGFB | vascular endothelial growth factor B | ENSG00000173511 |  |  |  |  |  |  |  |  |  |  |
| VEGFC | vascular endothelial growth factor C | ENSG00000150630 |  |  |  |  |  |  |  |  |  |  |
